# Reliable and valid robot-assisted assessments of hand proprioceptive, motor and sensorimotor impairments after stroke

**DOI:** 10.1186/s12984-021-00904-5

**Published:** 2021-07-16

**Authors:** Monika Zbytniewska, Christoph M. Kanzler, Lisa Jordan, Christian Salzmann, Joachim Liepert, Olivier Lambercy, Roger Gassert

**Affiliations:** 1 Rehabilitation Engineering Laboratory, Institute of Robotics and Intelligent Systems, Department of Health Sciences and Technology, ETH Zurich, Zurich, Switzerland; 2 Kliniken Schmieder Allensbach, Zum Tafelholz 8, 78476 Allensbach, Germany; 3 Future Health Technologies, Singapore-ETH Centre, Campus for Research Excellence And Technological Enterprise (CREATE), Singapore, Singapore

**Keywords:** Robot-assisted assessments, Neurorehabilitation, Stroke, Recovery, Somatosensation, Proprioception, Sensorimotor impairments, Hand function

## Abstract

**Background:**

Neurological injuries such as stroke often differentially impair hand motor and somatosensory function, as well as the interplay between the two, which leads to limitations in performing activities of daily living. However, it is challenging to identify which specific aspects of sensorimotor function are impaired based on conventional clinical assessments that are often insensitive and subjective. In this work we propose and validate a set of robot-assisted assessments aiming at disentangling hand proprioceptive from motor impairments, and capturing their interrelation (sensorimotor impairments).

**Methods:**

A battery of five complementary assessment tasks was implemented on a one degree-of-freedom end-effector robotic platform acting on the index finger metacarpophalangeal joint. Specifically, proprioceptive impairments were assessed using a position matching paradigm. Fast target reaching, range of motion and maximum fingertip force tasks characterized motor function deficits. Finally, sensorimotor impairments were assessed using a dexterous trajectory following task. Clinical feasibility (duration), reliability (intra-class correlation coefficient ICC, smallest real difference SRD) and validity (Kruskal-Wallis test, Spearman correlations $\rho$ with Fugl-Meyer Upper Limb Motor Assessment, kinesthetic Up-Down Test, Box & Block Test) of robotic tasks were evaluated with 36 sub-acute stroke subjects and 31 age-matched neurologically intact controls.

**Results:**

Eighty-three percent of stroke survivors with varied impairment severity (mild to severe) could complete all robotic tasks (duration: <15 min per tested hand). Further, the study demonstrated good to excellent reliability of the robotic tasks in the stroke population (ICC>0.7, SRD<30%), as well as discriminant validity, as indicated by significant differences (*p*-value<0.001) between stroke and control subjects. Concurrent validity was shown through moderate to strong correlations ($\rho$=0.4-0.8) between robotic outcome measures and clinical scales. Finally, robotic tasks targeting different deficits (motor, sensory) were not strongly correlated with each other ($\rho \le$0.32, *p*-value>0.1), thereby presenting complementary information about a patient’s impairment profile.

**Conclusions:**

The proposed robot-assisted assessments provide a clinically feasible, reliable, and valid approach to distinctly characterize impairments in hand proprioceptive and motor function, along with the interaction between the two. This opens new avenues to help unravel the contributions of unique aspects of sensorimotor function in post-stroke recovery, as well as to contribute to future developments towards personalized, assessment-driven therapies.

**Supplementary Information:**

The online version contains supplementary material available at 10.1186/s12984-021-00904-5.

## Background

At the level of the hand, somatosensory and motor function, as well as the interplay between the two, are essential for performing dexterous and skillful movements during activities of daily living (ADLs) [1–4]. For example when grasping a small object, proprioception is necessary to sense the current position of the limb [5, 6]. This sensory input is then integrated by the central nervous system to shape the motor output, a process called sensorimotor integration [7, 8]. Subsequently, the motor system is responsible for eliciting and executing the planned movement [9].

Neurological injuries such as stroke often disrupt specific aspects of this process, which consequently prevents affected individuals from performing ADLs [10, 11]. Often the exact impairments that cause activity limitations are unclear, although their detection would be a prerequisite to designing appropriate rehabilitation strategies tailored to each patient’s impairment profile [12]. Most commonly reported are motor impairments, with 80% of stroke survivors experiencing paresis [13–16]. However, some activity limitations that seem to originate from a motor function impairment may be caused by disturbed proprioceptive feedback [7]. Somatosensory function is in fact frequently affected and has been shown to be associated with poor functional recovery and higher activity limitations, although the reporting prevalence varies between 23 and 67% [17–22].

The difficulty in accurately identifying each patients’ impairment profile originates, among others, from the lack of sensitive assessment methods [23, 24]. Most widely used clinical assessments are observer-based and subjective, not optimal for providing reproducible stimuli, and prone to floor/ceiling effects [24, 25]. Further, many clinical methods focus on evaluating activity limitations (e.g. Action Research Arm Test, Box & Block Test [26, 27]), however there is a lack of tools that could help in understanding the underlying cause of decreased performance. Existing clinical assessments provide only a global measure of impairments (e.g. Fugl-Meyer Upper Limb Assessment [23]) and multiple assessments are needed to holistically evaluate sensorimotor impairment profiles, hence they are rarely performed at regular time intervals throughout rehabilitation [28]. As clinical methods typically do not assess somatosensory, motor and sensorimotor impairments through a single, standardized assessment setup, it is difficult to systematically compare those impairment modalities and understand how they change over time.

Technology-driven solutions provide a promising complement to conventional clinical assessments [1, 12, 29]. Robot-assisted methods are objective (not relying on observer judgement), accurate (e.g. able to measure exact body position/force applied), as well as capable of delivering precise, reproducible stimuli (e.g. to assess sensory function or spasticity [30, 31]). Further, it becomes possible to evaluate different impairments with one single device through multiple robot-assisted assessment tasks, which results in a time-efficient and more comprehensive overview of impairments. This also allows to compare different impairment modalities (e.g. motor and sensory) with each other in a standardized way, potentially providing new insights into upper limb impairment profiles. Even though they are promising, the existing robotic approaches aiming at concurrent sensory and motor assessment of the hand remain in their infancy. The methods proposed so far focus on proximal joints of the upper limb [32, 33], consist of tasks that target only a specific impairment modality (e.g. proprioception, without the possibility to concurrently assess motor impairment) [34–37], or fail to provide a detailed evaluation of clinimetric properties of their outcome measures (reliability, measurement error, validity) [12, 38]. Reporting of test-retest reliability and measurement error is essential to understand the sensitivity of an assessment metric to capture different impairments and detect changes over time [38], while the study of concurrent validity is important to relate a new technological approach to the commonly accepted assessment methods [39]. The current lack of standardized evaluations of reliability and validity in the target population makes new assessment technologies less likely to be clinically accepted and applied outside of research projects [40].

The objective of this work was to propose and evaluate a new set of assessments of hand proprioceptive, motor and sensorimotor impairments, implemented on a single, previously described robotic platform (ETH MIKE: Motor Impairment and Kinesthetic Evaluation) [41, 42]. This one degree-of-freedom end-effector device can provide well-controlled movement stimuli to the index finger metacarpophalangeal (MCP) joint and sensitively measure its kinematic and kinetic responses. The index finger was selected due to its relevance in many ADLs (grasping, precision grip [43]). Furthermore, the ability to actively extend the MCP joint is often presented as an early predictor of functional recovery, as it is related to the degree of sparing of cortico-motoneuronal pathways after stroke [44, 45]. From a practical perspective, focusing on a single joint allows to simplify the technology, which increases clinical usability. In this paper we propose a battery of five behavioural tasks and their outcome measures, three of which address motor impairments, one targets proprioception and one measures combined sensorimotor deficits. We investigate the reliability and validity of these robot-assisted assessments in a group of 36 participants with stroke and in an age-matched group of 31 neurologically intact controls. We hypothesized that the newly proposed robot-assisted assessment metrics (i) are reliable due to the objective nature of the tasks, their repeatability and the standardized protocol; (ii) allow to distinguish stroke patients from control subjects and identify different impairment profiles; (iii) can separately quantify proprioceptive, motor and sensorimotor impairments and correlate with corresponding clinical scales.

This work aspires to contribute to the field of neurorehabilitation by providing novel objective assessments, which aim at disentangling different aspects of sensorimotor impairments in order to better understand the cause of observed activity limitations. In the long term the proposed robot-assisted assessments intend to help in designing more effective therapies, as well as in tracking and predicting recovery of patients after neurological injuries.

## Methods

### Subjects

Thirty-six participants with stroke were recruited for this study among the patients receiving an inpatient neurological rehabilitation at the Kliniken Schmieder Allensbach, Germany. Inclusion criteria were: above 18 years old, diagnosis of stroke (ischemic or hemorrhagic), and the ability to passively move the subject’s MCP joint by at least $20^{\circ }$. Exclusion criteria were: inability to understand instructions and pain when moving the MCP joint. Moreover, we designed the study to include a maximum of 40% of subjects with intact proprioception as measured by a conventional clinical scale. This design choice was made to allow for validating the newly proposed measure of proprioception. In addition, thirty-one age-matched neurologically intact control subjects were recruited. The inclusion criteria for this group were: right-handed and above 50 years old. The exclusion criteria was any history of neurological, orthopaedic or rheumatologic disease affecting wrist or hand function. In both groups, handedness was assessed using the Edinburgh Handedness Inventory, where stroke subjects were asked to evaluate their pre-stroke handedness retrospectively. All subjects gave written informed consent before participating in the experiment. The study was approved by the ETH Ethics Committee EK 2019-N-108 and the Ethics Commission of Baden-Württemberg F-2016-126 and retrospectively registered as a clinical trial^1^.

### Robot-assisted assessments

#### Apparatus

**Fig. 1:**
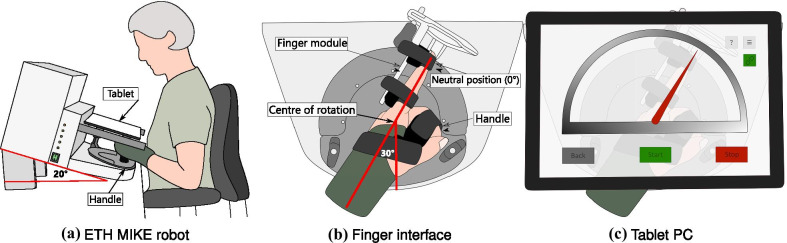
Schematic of the robotic platform ETH MIKE. **a** Subjects are seated in front of the ETH MIKE robot, with their elbow supported on an arm rest. A wrist splint is worn to avoid any compensatory movements at the wrist. The device is inclined by $20^{\circ }$ to minimize parallax errors.** b** The hand is wrapped around a handle, which is set up at the wrist neutral position ($0^{\circ }$ wrist flexion, $30^{\circ }$ from the middle of the device’s workspace), ensuring a comfortable resting position for the wrist. The index finger is attached to an adjustable finger module by Velcro straps. The centre of rotation of the end-effector is aligned with the MCP joint. **c** A tablet computer is placed above the hand, removing visual cues from the tested hand and providing an interactive graphical user interface displaying a simple gauge with a red indicator

The ETH MIKE (Motor Impairment and Kinesthetic Evaluation)^2^ is a one degree of freedom end-effector robot, which can provide well-controlled stimuli to the index finger and sensitively measure subjects’ kinematic and kinetic responses [41, 42]. The end-effector has its center of rotation aligned with the MCP joint of the index finger. Subjects are seated in front of the device, the hand is placed grasping an easily exchangeable, 3D printed handle, and the index finger is stretched and attached to the end-effector via Velcro straps (Fig. 1a). For a natural and comfortable positioning, the hand of the subject is placed in the device with a $30^{\circ }$ angle from the middle of the end-effector’s workspace (Fig. 1b, c). The device is suitable to test both hands, one hand at a time. A tablet computer with a touch screen is placed directly above the hand, displaying a Graphical User Interface (GUI) programmed in Unity (Unity Technologies, California, USA), that is used as a visual display during the assessment tasks. To minimize cognitive load, the GUI displays a simple gauge with colored indicators for all assessment tasks (Fig. 1c).

The hardware of the robot consists of one actuator (a DC motor), as well as an incremental encoder, a tachometer and a force sensor. The device is controlled by a real-time embedded board (myRIO, National Instruments, Texas, USA) and programmed in LabVIEW (National Instruments, Texas, USA). The end-effector position, velocity and interaction force signals are recorded at a sampling frequency of 1 kHz. Velocity and force signals are smoothed in real-time using a 1st order Butterworth low-pass filter with 20 Hz cutoff frequency. Post-processing is implemented in MATLAB (Mathworks Inc., Massachusetts, USA).

#### Robotic assessment battery

The battery of robot-assisted assessments consists of tasks targeting proprioceptive and motor impairments, as well as the interplay between the two (sensorimotor impairments). Specifically, there is one task for proprioception assessment (gauge position matching), three tasks focused on motor impairments (range of motion, maximum force generation and fast target reaching). Additionally, one task is designed to evaluate the ability to integrate proprioceptive information to execute a complex movement, i.e. sensorimotor impairments (trajectory following). All of these tasks are performed on the robotic platform ETH MIKE. A motivation from related literature, the task procedures, and sensor-based metrics extracted from each task are described below.

**Fig. 2:**
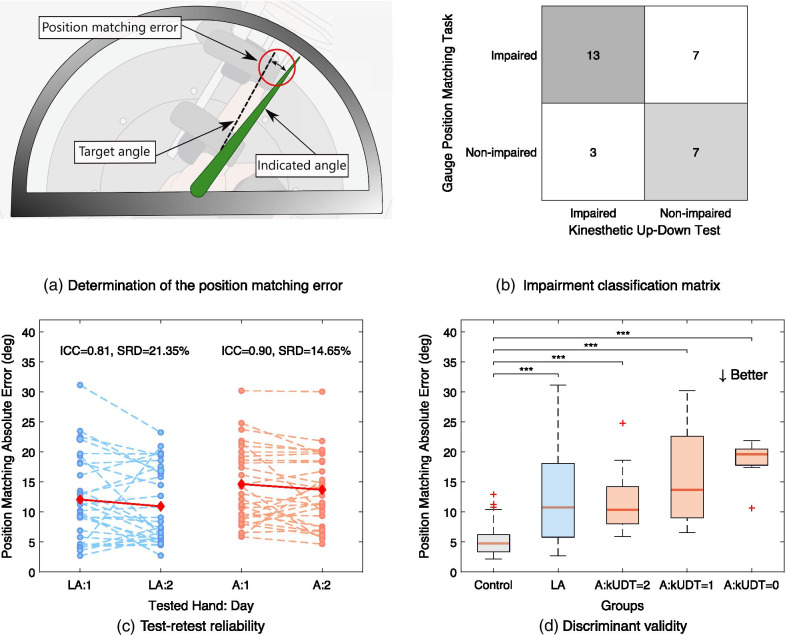
Gauge position matching task for the assessment of proprioceptive impairments. **a** The index finger is first passively moved to a target angle, and the subject then needs to indicate the perceived finger position on the tablet screen (no visual feedback is provided). The outcome measure is the error between indicated angle and target angle. Note that on the Fig. the screen is shown as semi-transparent for illustration purpose. During the assessment, patients cannot see the assessed finger, which is located underneath the tablet. **b** There is a high agreement (70%) in impairment classification between the gauge position matching task metric and the clinical measure of proprioception (kUDT). **c** The task metric has good (ICC$>0.75$) reliability on the less affected and excellent reliability (ICC$>0.90$) on the affected side. **d** The box plot indicates a trend of increasing position matching absolute error with increasing proprioceptive impairment (according to kUDT). There is a significant difference between control (N=62) and all stroke impaired sub-groups (kUDT=2: N=14, kUDT=1: N=8, kUDT=0: N=8).* Acronyms - LA:* less affected, *A:* affected, *kUDT:* kinesthetic Up-Down Test, *ICC:* intraclass correlation coefficient, *SRD*: smallest real difference. Statistical significance: *p*-value<0.05: *, *p*-value<0.01: **, *p*-value<0.001: ***

**Gauge position matching task—assessment of proprioceptive impairments**: the objective of this assessment is to evaluate the MCP joint proprioception, while minimizing possible confounds coming from motor impairments. The task is based on previous studies that optimized the gauge position matching task procedure [46, 47]. Compared to a 2 alternative forced choice paradigm often used to evaluate somatosensory function [36, 48], the gauge position matching task is faster and does not rely on subjects remembering and comparing positions. The task procedure is the following: after the tested finger has been passively moved to a target angle by the robot, the user is prompted to indicate the perceived finger position on the tablet screen, located directly above the hand, by moving a virtual gauge indicator to a position aligned with the tip of the tested index finger (Fig. 2a). Their view of the hand is constrained by the location of the tablet, hence subjects can not compensate by visual feedback to complete the task. Every trial starts with the robot moving the finger from the neutral position ($0^{\circ }$ angle at the MCP joint) to one of 21 angles (integer values [$10-30^{\circ }$] in flexion from the neutral MCP joint position) within 3 seconds. In one assessment, each angle is presented once, in a random order. Previous work has shown that sampling each angle once is sufficient to reliably assess proprioception, while minimizing the duration of the test [47]. There is no time constraint for the subjects to indicate the perceived position and no feedback is given about the subject’s performance. To ensure that the task assesses one hand only and does not rely on subject’s ability to indicate the perceived position on the screen with the other hand, the experimenter helps the subject to point to the perceived finger position on the screen. For all stroke and control subjects, the experimenter first asks if the gauge indicator on the tablet screen is below or above the reference position and then moves the gauge indicator slowly in that direction, by dragging it on the touch screen, until the subject says “stop”. Then the experimenter asks for confirmation and allows for final adjustments. For each trial, the absolute error is calculated by taking the absolute value of the difference between the reported and the presented angle. The primary outcome measure is the mean value of this absolute error across all 21 trials, denoted *Position Matching Absolute Error*. The higher the absolute error, the worse the task performance.

**Fig. 3:**
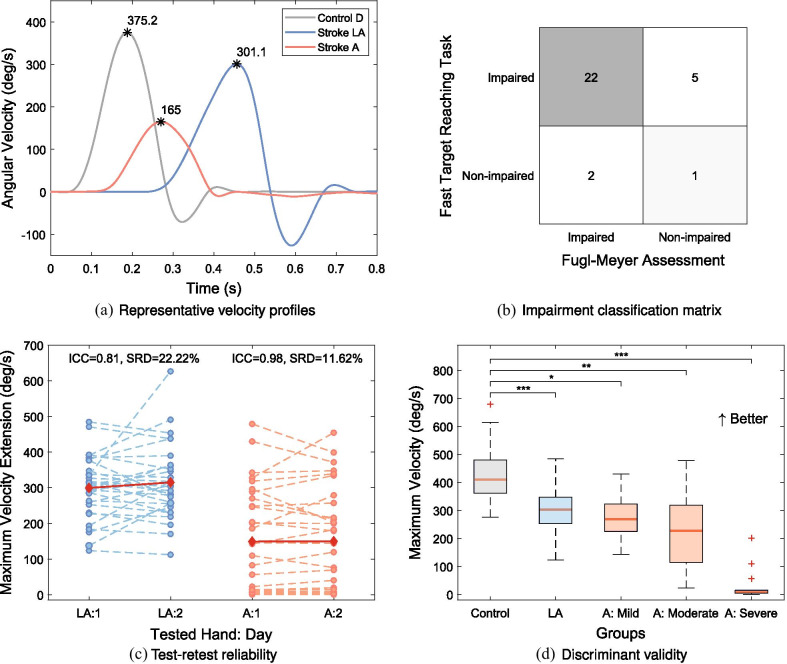
Fast target reaching (extension) task for the assessment of motor impairments. **a** Subject are instructed to move their index finger as fast as possible from a starting position (30$^{\circ }$ flexion from the MCP joint neutral position) to a target position (10$^{\circ }$ extension from the MCP joint neutral position) in a single, ballistic movement. The outcome measure is the maximum velocity reached during that movement. **b** The task has good reliability (ICC$> 0.70$) on the less affected and excellent reliability (ICC $>0.90$) on the more affected side. **c** There is a high agreement (80%) in impairment classification between the task metric and the clinical measure of upper-limb motor impairments FMA. **d** The box plot indicates a tendency of decreasing robotic task performance with increasing stroke severity (mild: FMA$>=54$, moderate: $54>$FMA$>=35$, severe: FMA$<35$). There is a significant difference between control (N=62) and all stroke sub-groups—severe (N=14), moderate (N=8) and mild (N=8). Acronyms - *D: *dominant,* LA:* less affected, *A:* affected, *FMA:* Fugl-Meyer Upper Limb Motor Assessment, * ICC:* intraclass correlation coefficient, *SRD:* smallest real difference. Statistical significance: *p*-value<0.05: *, *p*-value<0.01: **, *p*-value<0.001: ***

**Fast target reaching task—assessment of motor impairments (1)**: the objective of this task is to quantify subjects’ ability to produce fast ballistic target reaching movements. Target reaching has been used before as an assessment method of motor function deficits [49–52]. However, in contrast to target reaching tasks typically implemented in literature [49–52], in the newly proposed task the velocity is of interest and the accuracy of the movement is not considered. We designed the task in a way to minimize the involvement of somatosensory feedback in the movement generation, thereby relying on feedforward control. Subjects are instructed to move as fast as possible, in a single movement, from a starting position to a target, each displayed on the tablet computer screen as a red and green gauge indicator respectively. We therefore expect a ballistic movement, with minimal end-point correction since no visual feedback on the current position is provided and the finger is hidden under the tablet. The movement is performed either in flexion or in extension direction, in a random order. First, the tested finger is passively moved to a starting position by the robot ($-10^{\circ }$ from neutral joint angle as starting position for flexion and $30^{\circ }$ for extension trials). Then, after a 3-second countdown, subjects are instructed to move as fast as possible to the target (displayed at $30^{\circ }$ for flexion and at $-10^{\circ }$ for extension trials). Four seconds are given for all subjects to move to the target, which was chosen to standardize the protocol and ensure that subjects with a slower reaction have enough time to generate a movement. Subjects are instructed to remain at their position once they believe they have reached the target. One assessment consists of 20 trials (10 times each direction). The primary outcome measure is the mean of the three maximum velocity values (in $^{\circ }/s$) over all 10 trials per movement direction (denoted *Maximum Velocity Flexion/Extension*). The higher the velocity, the better the task performance. Representative velocity profiles are shown in Fig. 3a.

**Range of motion task—assessment of motor impairments (2)**: the purpose of this task is to measure the range of motion of the index finger in flexion and extension direction. The range of motion is regularly evaluated in clinical settings to describe hand impairments [28, 53, 54]. Stroke subjects often show limited range of motion and the ability to extend the finger early post-stroke has even been shown as a predictor of recovery [45]. In this task subjects are instructed to move the index finger (which is secured on the ETH MIKE finger interface) as far as possible first in flexion and then in extension direction. This is repeated three times. Subjects can see the visual feedback of their finger displayed on the tablet computer. Afterwards, the same task is repeated in a passive manner, meaning that the experimenter moves the subject’s finger in flexion (until the end of the range of motion of the robot or until the subject says “stop” due to discomfort) and then in extension (until the experimenter detects tension in subject’s finger by feeling some resistance against the movement or until the subject says “stop”), while the subject is instructed to relax his/her finger. Here, the tablet computer is removed so as to not obstruct the experimenter that induced the motion. For each repetition, the difference between the maximum position in flexion and the maximum position in extension (measured in degrees) is calculated (denoted as *Active/Passive Range of Motion – AROM/PROM*). The primary outcome measure is the mean value across three repetitions for both AROM and PROM. The higher the ROM, the better the task performance. Representative position profiles are shown in Additional file 1: Fig. SM1a.

**Maximum fingertip force generation task—assessment of motor impairments (3)**: the objective of this task is to measure maximum fingertip force. Assessments of grip strength are often performed in clinical settings in patients after stroke [55, 56], as weakness is frequently present after stroke and is linked to the damage to the corticospinal tract [13]. In this task procedure the end-effector is first blocked by a fixation mechanism, located at a $15^{\circ }$ flexion angle at the MCP joint (with respect to a neutral position where all phalanges are aligned). The subjects are instructed to generate maximal force with their index finger for an indicated period of time (3 s), preceded by a 3 s preparation phase. No verbal or visual feedback related to the magnitude of the generated force is provided to the participants during the task. Three repetitions are performed first in flexion and then in extension direction. The primary task metric is the mean of the maximum force over three trials for both the flexion and extension direction, measured in Newtons by the force sensor located at the end-effector (denoted *Maximum Force Flexion/Extension*). The higher the force, the better the task performance. Representative force profiles are shown in Additional file 1: Fig. SM2a.

**Fig. 4:**
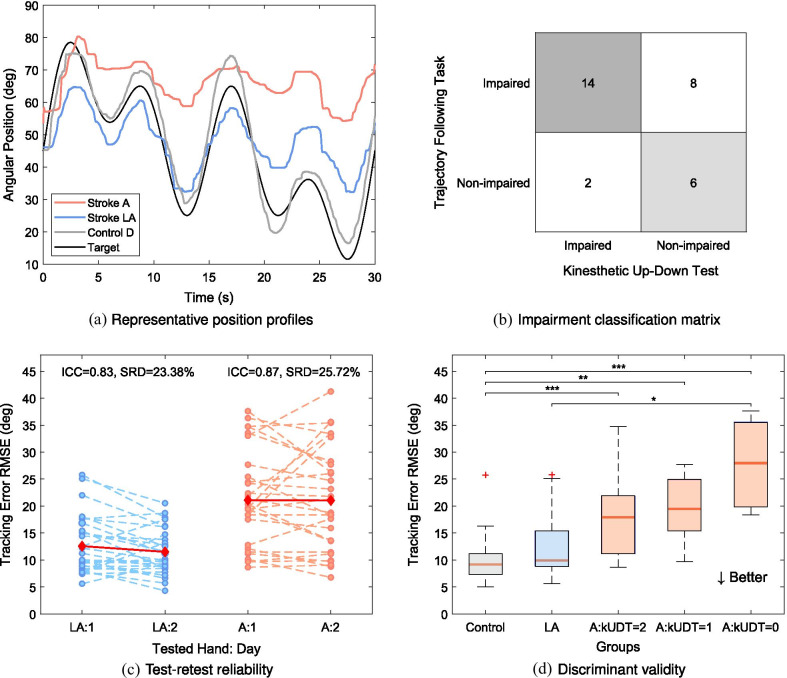
Trajectory following (slow) task for the assessment of sensorimotor impairments. **a** Subjects need to actively follow with their index finger a target trajectory displayed on the tablet screen. Control subjects can follow the target more accurately, while stroke subjects are further away from the target and their movement is more fragmented. **b** There is a high agreement (70%) in impairment classification between this task metric and the clinical measure of proprioception (kUDT). **c** The task has good reliability on both the less affected and on the more affected side (ICC $>0.70$). **d** The box plot indicates increasing tracking error RMSE with increasing proprioceptive impairment (according to kUDT). There is a significant difference between controls (N=62) and all stroke sub-groups on the affected side (kUDT=2: N=14, kUDT=1: N=8, kUDT=0: N=8), as well as between less affected side of stroke subjects (N=30) and the group with the most severely impaired proprioception (N=8). Acronyms - *D:* dominant, *LA:* less affected, *A:* affected, *kUDT:* kinesthetic Up-Down Test, *ICC:* intraclass correlation coefficient, *SRD:* smallest real difference. Statistical significance: *p*-value<0.05: *, *p*-value<0.01: **, *p*-value<0.001: ***

**Trajectory following task—assessment of sensorimotor impairments**: the aim of this task is to assess finger dexterity, which relies both on proprioceptive function and motor execution. Trajectory following has been used previously to evaluate fine motor control [57–59]. First, the index finger is passively moved to a starting position by the robot ($15^{\circ }$ flexion angle at the MCP joint). After a three second countdown, a trajectory is displayed on the tablet screen in the form of a moving gauge indicator, which the subjects are instructed to follow as accurately as possible. The vision of the actual finger position is not displayed on the screen, to ensure that subjects rely on proprioception to guide the motion. Two trajectory scenarios are displayed (slow and fast) in order to diversify the task. Each trajectory consists of three superimposed sine waves, each of different frequency and the same amplitude ($15^{\circ }$). The slow trajectory consists of the following sine wave frequencies: 0.03 Hz, 0.07 Hz and 0.13 Hz, while the fast trajectory is composed of 0.10 Hz, 0.20 Hz and 0.40 Hz. One trial lasts 30 seconds and in total there are six trials in one assessment (three times each trajectory, first 3 times slow, then 3 times fast). For each trial, the tracking error between the trajectory displayed on the screen and the performed motion is calculated (Root Mean Squared Error RMSE [57]). The primary outcome measure is the mean across the three trials for the slow and the fast trajectory (denoted *Tracking Error RMSE Slow/Fast*). The higher the tracking error, the worse the task performance. Representative trajectories are shown in Fig. 4a.

### Clinical assessments

The following clinical assessments were performed by a trained physiotherapist as a part of the study protocol. The kinesthetic Up-Down Test (kUDT) as part of the Nottingham Sensory Assessment (NSA) was chosen as a measure of proprioception (performed with the forearm fully pronated and the wrist in a neutral position) [60]. In order to keep the scoring system of the kUDT from the NSA consistent with the commonly used Erasmus modified Nottingham Sensory Assessment [61], scores 1 and 2 were grouped together as score 1 and the best score was assigned the value 2. To clinically evaluate motor impairments, the Fugl-Meyer Upper Limb Motor Assessment (FMA) was used [23]. The Box & Block Test of Manual Dexterity (BBT) was selected as an assessment of combined sensorimotor function and activity limitations [27] and it was completed for both hands. To quantify cognitive function, the Montreal Cognitive Assessment (MoCA) was performed [62]. Finally, the Modified Ashworth Scale (MAS), performed at the MCP joint of the index finger, was used as a measure of spasticity [63].

### Experimental protocol

Two testing sessions on two separate days were conducted by the same experimenter to evaluate test-retest reliability of robotic task metrics in stroke subjects. Clinical assessments were performed in a separate session. For the control subjects, the protocol consisted of only one experimental session with the robot.

Subjects were seated in front of the robotic device and the height of the chair and the armrests was adjusted to a comfortable seating position close to the robot (Fig. 1a). A wrist splint was used to ensure that the MCP joint was tested in isolation without any compensatory movements from the wrist. The elbow of the subjects was placed on the cushioned armrest and subjects were instructed to keep it close to their body and to avoid compensatory movements throughout the trial. The hand was strapped to the handle after ensuring optimal alignment of the forearm and the wrist joint with the orientation of the handle (neutral position of the wrist, $30^{\circ }$ from the middle of the device workspace—Fig. 1b). The index finger was attached to the finger module. The robotic assessments were always started with the range of motion and maximum fingertip force generation tasks, as they were the least complex and helped subjects to get familiar with the device. The order of the other three tasks, as well as the starting hand were randomized. Afterwards, subjects performed the assessments in the same order with the other hand. There was a familiarization round before each task. It consisted of a shortened version of the task, with only half the number of trials, and where subjects were instructed and encouraged to ask any questions they may have related to the task.

### Data analysis

#### Descriptive statistics

The metrics of the robot-assisted assessments are reported as mean and standard deviation per hand per studied group. In order to analyze potential confounding effects on task outcome measures emerging from subject demographics and task protocol, linear mixed effect models (LME) were built based on the control subjects dataset, following the steps defined in Kanzler et al. [39]. The parameters suspected to have a confounding effect on robotic metrics and hence included in the model were age, gender, tested hand (left/right) and the task specific effect. The latter was considered since some of the robot-assisted tasks were performed multiple times under different conditions, for example in two directions (e.g. maximum force in flexion/extension) or at different speeds (e.g. slow/fast trajectory following). The statistical significance of these effects was tested using the t-statistic (significance level of 0.05). In addition, the ability of the models to represent the experimental data (model quality) was analyzed according to the criteria C1 and C2, which characterize the mean absolute error of the model and its variability [39, 64] (moderate quality: C1 $\le$ 15% and C2 $\le$ 25%; good quality: C1 $\le$ 10% and C2 $\le$ 20%).

#### Test-retest reliability

For a comprehensive evaluation of the reliability of the newly proposed robot-assisted assessments, different statistical measures were considered. First, the intraclass correlation coefficient ICC(A,k) was used to calculate absolute agreement between test and retest based on a two-way analysis of variance, taking into account all individual trials on test and retest [39, 65, 66]. This statistical method characterizes how well it is possible to discriminate between subjects across testing days (taking into consideration inter-subject and intra-subject variability). Acceptable ICC values are above 0.7 [39, 67]. Secondly, smallest real difference (SRD) and SRD% (% with respect to the range across all trials of a task) were calculated. These measures describe how well it is possible to distinguish between measurement noise and an actual physiological change [68]. Previous work suggested a cut-off of 30% for the SRD% to identify metrics without strong measurement error [39]. Further, to identify potential learning effects, the presence of possible systematic shifts between test and retest was analyzed. This was expressed as a mean difference between test and retest normalized with respect to the range of observed values [39]. Previous work suggested to consider a range of systematic shifts of [-6.35 and 6.35] to identify metrics without strong learning effects [39]. Bland-Altman plots were used as an alternative check for systematic bias [69]. Finally, as a general check of similarity between test and retest, Spearman rank-order correlations between test and retest were calculated. It is desired for the test and retest metrics for each task to be strongly correlated, since that shows that the task outcomes are comparable between test and retest.

#### Discriminant validity

In order to define if the robotic metrics are capable of capturing abnormal task performance and thus impairments, the task metrics were compared between the stroke and the control group. This comparison was performed using three statistical methods. Firstly, control subjects were compared to the affected and the less-affected side of stroke subjects using the Kruskal-Wallis test (Bonferroni corrected). The same group comparison was also performed using the Area Under the Curve (AUC) of the Receiver Operating Characteristic [39]. This method defines true positive/true negative rates of classifying subjects into two groups (stroke/control). A metric can well discriminate between the two groups if AUC is above 0.7 [39]. Finally, z-score normalization was implemented to find the percentage of stroke subjects performing worse than the 95th percentile of control subjects, thereby allowing to identify individuals that are impaired according to a specific outcome measure. To avoid comparing, for example, maximum fingertip force generated by an older female to a control population that is on average younger and gender-mixed, potentially confounding effects were removed based on the LME analysis proposed by Kanzler et al. [39]. Specifically, the effect of age, gender, tested hand, trial number, as well as the task-specific effect (e.g. the effect of movement direction for the fast target reaching task) on the outcome measures of the ETH MIKE were removed. The removal of these potentially confounding effects is essential to avoid bias when comparing data from patients with a control population. This procedure was only implemented for the z-score normalized robotic metrics within the motor category, as only these metrics were found to be significantly affected by the majority of the identified confounds (Additional file 1: Table SM1). The less and the more affected sides of stroke subjects were compared using AUC (group level comparison) and a paired-sample t-test (per-subject comparison between the body sides).

#### Concurrent validity

To determine if the newly proposed tasks are able to capture impairments they were designed to assess, each task outcome measure was correlated with the clinical score that was expected to best reflect the underlying physiological construct. Namely, Spearman correlation was used to find the relationship between each task metric and the three clinical assessments: BBT, FMA and kUDT. The correlation strength was defined as: $\rho <0.1$ negligible, $0.1<\rho <0.39$ weak, $0.4<\rho <0.69$ moderate, $\rho >0.7$ strong [70]. Further, the classified impairments based on the ETH MIKE metrics were compared to the impairments detected by the corresponding clinical scores. This was implemented on per-subject basis, leading to an overall agreement between the subjects classified as impaired according to the robotic and the clinical measures. The clinical scores used for classifications were FMA and kUDT, as both of these measures have clearly defined impairment classification thresholds (impaired defined as FM $< 60$ [71] and kUDT $< 2$ [61]).

#### Independence of task outcome measures

Partial Spearman correlation was calculated between the outcome measures of tasks that aim to characterize different impairments (proprioceptive, motor, sensorimotor), in order to determine if the proposed battery of tasks presents complementary information. If a task consisted of two metrics (e.g. Maximum Velocity Flexion and Extension in the fast target reaching task), only the one with the highest reliability and validity was chosen for this analysis. This is motivated by the fact that, by design, the two metrics within the same task are likely correlated, while the objective of this analysis was to find possible relationships between different domains (e.g. fast target reaching and gauge position matching).

## Results

Out of the recruited 36 subjects with stroke, 34 successfully completed the two sessions of robot-assisted assessments. Two stroke subjects dropped out of the study (ID 10 and 20—Additional file 1: Table SM4), because they both felt fatigued/unwell during the first robotic assessment session and decided not to participate in the second session. Additionally, three participants (ID 4, 23 and 31) had missing data in one of the robotic assessments. The reason was either data saving malfunction or because they had another urgent appointment in the clinic and the full robotic assessment protocol could not be completed. Finally, one subject (ID 9) had severe problems with task comprehension and was not able to correctly follow the robotic task instructions. Altogether 30 stroke subjects were included in the data analysis, aged $64.50 \pm 14.02$ years, 19 males, 4 left-handed before stroke, 12 with left hemispheric stroke, 21 with ischemic and 9 with hemorrhagic stroke. All except one (chronic, 98 weeks post-stroke) were sub-acute ($8.17 \pm 4.56$ weeks post-stroke), as summarized in Table 1. The number of days between test and retest was $1.97 \pm 0.18$. The group of stroke subjects that completed the protocol had a diverse range of impairments, from severe (FMA=4 and kUDT=0) to mild (FMA=65, kUDT=3). The average result of FMA was $34.43 \pm 22.22$ and of kUDT $1.20 \pm 0.83$. The time to perform the robotic assessment protocol (excluding instructions and setup) was $13.74 \pm 4.55$ min on test and $12.34 \pm 2.98$ min on retest on the affected side (details in Tables 3 & SM3). It took a comparable amount of time to complete the tasks on the less affected side ($14.09 \pm 3.32$ min test and $13.03 \pm 2.30$ min retest). Thirty-one age-matched control subjects were recruited in this study. None were excluded from the statistical analysis. Their average age was $66.87 \pm 7.92$ years, 20 were male and all were right-handed (Table 1).

Descriptive statistics results (mean and standard deviation) of each task metric for the affected side are reported in Table 2, for the less affected side in Additional file 1: Table SM2 and for control subjects in Additional file 1: Table SM1. The outcomes of the LME are shown in Additional file 1: Table SM1. Overall, model quality was moderate to good for all tasks except for the position matching task. The maximum fingertip force generation task was significantly affected by age (t=$-2.83$, DF=366, *p*-value=0.0049). The outcome measure of that task was also affected by gender (t=$-5.00$, *p*-value<0.001), which was also a confounding effect for the fast target reaching task (t=$-3.73$, *p*-value<0.001). The effect of the tested hand was significant for all three tasks within the motor impairment category. All metrics were significantly influenced by the task-specific effect. Most interestingly, the position matching error was influenced by the presented angle magnitude. The larger the angle to which the subject’s finger was passively moved, the larger was the matching error.

**Table 1:** Participants information

	Stroke subjects	Control subjects
N	30	31
Age (mean ± SD)	64.50 ± 14.02	66.87 ± 7.92
Male $\mid$ Female	19 $\mid$ 11	20 $\mid$ 11
Handedness	4 Left	Right only
LHS $\mid$ RHS	12 $\mid$ 18	
Ischemic $\mid$ Hemorrhagic	21 $\mid$ 9	
Weeks since stroke	8.17 ± 4.56 (range [2.57, 20.86])	
FMA [0-66]	34.43 ± 22.22 ([4, 65])	
kUDT [0-2]	1.20 ± 0.83 ([0, 2])	
BBT affected side [#/min]	20.90 ± 20.16 ([0, 74])	
BBT less affected side [#/min]	60.30 ± 11.27 ([44, 80])	
MoCA [0-30]	22.00 ± 5.84 ([6, 30])	
MAS [0-6]	0.10 ± 0.55 ([0, 3])	

These results only consider subjects that were included in the data analysis (i.e. does not consider subjects that were excluded due to missing data, drop-outs etc.). Acronyms—LHS: Left Hemispheric Stroke; RHS: Right Hemispheric Stroke; FMA: Fugl-Meyer Upper Limb Motor Assessment; kUDT: kinesthetic Up-Down Test; BBT: Box& Block Test; MoCA: Montral Cognitive Assessment; MAS: Modified Ashworth Scale

### Test-retest reliability

All task metrics had sufficient test-retest reliability according to the defined criteria for the affected side of stroke subjects (Table 2). Specifically, ICC was good to excellent (ranging from 0.86 for Tracking Error RMSE Fast to 0.98 for Maximum Velocity Extension), the measurement error was small (SRD%<30% for all tasks) and the systematic shift was within the defined range (the smallest value of $-3.63$ for Position Matching Absolute Error was still above the threshold of $-6.35$). Similarly, no systematic bias was detected in Bland-Altman plots, as the datapoints were equally distributed above and below the mean (Additional file 1: Fig. SM3–SM7). Test and retest were strongly correlated for all tasks on the affected side ($\rho$ ranging from 0.74 to 0.97). On the less-affected side, ICC and SRD% were within required thresholds for all task metrics except for Maximum Velocity Flexion (ICC=0.59, SRD%=40.40%), as summarized in Additional file 1: Table SM3. Further, the systematic shift was equal to 7.27 for Maximum Force Extension and $-7.24$ for Tracking Error RMSE Fast. It was also possible to observe a negative shift in the mean difference between test and retest scores in the Bland-Altman plots for these two tasks (Additional file 1: Figs. SM6, SM7). For all other tasks, systematic shift was within $\pm 6.35$. There was a moderate to strong significant correlation between test and retest for all tasks on the less affected side ($\rho$ ranging from 0.44 to 0.78). Graphical representations of the test-retest reliability of one example task per category (proprioception, motor and sensorimotor) for both affected and less affected sides are shown in Figs. 2c–4c.

**Table 2:** Test-retest reliability results for all task metrics on the affected side

Category	Metric	Mean±SD T1	Mean±SD T2	Mean±SD T2-T1	ICC(A,k)	SRD	SRD(%)	Shift	Correlation
Sensory	AE [*deg*]	14.63 ± 6.43	13.71 ± 6.24	2.68 ± 2.94	0.90 (0.88–0.91)	9.12	14.65	-3.63	0.74***
Motor	Vel. Flex. [*deg*/*s*]	314.94 ± 180.30	315.16 ± 162.50	0.22 ± 107.56	0.89 (0.84-0.93)	159.54	17.83	-0.003	0.84***
	Vel. Ext. [*deg*/*s*]	149.06 ± 146.27	149.80 ± 144.74	0.74 ± 43.80	0.98 (0.97-0.98)	60.68	11.62	-0.15	0.97***
	AROM [*deg*]	43.89 ± 36.20	42.11 ± 32.29	-1.78 ± 11.16	0.97 (0.96–0.98)	15.58	12.77	1.47	0.92***
	PROM [*deg*]	83.88 ± 10.48	85.41 ± 7.95	1.53 ± 5.89	0.89 (0.83–0.93)	8.83	16.57	-3.02	0.83***
	Force Flex. [*N*]	11.64 ± 11.55	10.72 ± 10.08	-0.91 ± 3.44	0.97 (0.96–0.98)	4.88	10.92	2.07	0.94***
	Force Ext. [*N*]	4.12 ± 4.52	3.55 ± 3.67	-0.57 ± 1.93	0.94 (0.91–0.96)	2.80	16.72	3.80	0.93***
Sensorimotor	RMSE Slow [*deg*]	21.09 ± 8.68	21.03 ± 9.40	-0.06 ± 6.12	0.87 (0.81–0.92)	9.19	25.72	-0.17	0.81***
	RMSE Fast [*deg*]	21.45 ± 7.80	22.06 ± 9.06	0.60 ± 5.87	0.86 (0.79–0.91)	8.82	24.75	1.95	0.82***

*AE:* Position Matching Absolute Error, *Vel. Flex./Ext.:* Maximum Velocity Flexion/Extension, *AROM/PROM:* Active/Passive Range of Motion, *Force Flex./Ext.:* Maximum Force Flexion/Extension, *RMSE Slow/Fast:* Tracking Error RMSE Slow/Fast, *T1:* Test, *T2:* Retest, *ICC:* Intraclass Correlation Coefficient, *SRD:* Smallest Real Difference, *SRD%:* Smallest Real Difference as a percentage of the range of values across all trials, *Shift:* Systematic Shift, * Correlation:* Spearman Correlation between Test and Retest. The defined thresholds for reliability were: ICC>0.70, SRD%<30%, Shift$<\mid$6.35$\mid$ (where negative shift means improvement on retest and positive means worse performance on retest). Statistical significance was defined as: *p*-value<0.05: *, *p*-value<0.01: **, *p*-value<0.001: ***

### Discriminant and concurrent validity

Summary results of discriminant and concurrent validity for each task metric are shown in Tables 3 (for the more affected side) & SM3 (for the less affected side). A graphical representation of group differences (with severity subgroups within the affected side of stroke subjects) are shown for one outcome measure per task in Figs. 2d–4d & SM1d-2d. Details on the classification agreement are provided in the form of a matrix (Figs. 2b–4b & SM1b-2b).

Position Matching Absolute Error allowed to discriminate between controls and the affected side of stroke subjects (*p*-value<0.001; AUC=0.95), as well as between controls and the less-affected side of stroke subjects (*p*-value<0.001; AUC=0.82). There was no significant difference between the affected and the less affected side of stroke subjects (t-stat=1.84, *p*-value$=$0.077; AUC=0.61). 66.67% of subjects were classified as impaired on their affected and 56.67% on their less affected side. Position Matching Absolute Error correlated moderately with the clinical measure of proprioception ($\rho$=$-0.48$, *p*-value=0.007). Further, there was a weak significant correlation between the robotic task score and the BBT ($\rho$=$-0.37$, *p*-value=0.046) and no significant correlation with the FMA. The agreement between impairment classification by robotic and clinical measure was 70.00%.

There was a significant difference between control group and the affected side of stroke subjects for all task outcome measures within the motor impairments assessment category, although Passive Range of Motion AUC was just below the threshold (*p*-value=0.007; AUC=0.69). Further, on a group-level stroke subjects performed significantly worse also on their less affected side comparing to controls in the fast target reaching task (Flexion: *p*-value<0.001; AUC=0.85, Extension: *p*-value<0.001; AUC=0.85). Additionally, all task outcomes within motor category, except for Maximum Velocity Flexion (t-stat=1.93, *p*-value=0.064; AUC=0.62), could discriminate between the less and the more affected sides of stroke subjects. Across all motor assessment tasks, 23.33-90.00% of subjects were classified as impaired on their affected side, and 3.33-60.00% on the less affected side. Strong significant correlations were found between all tasks within the motor category and the FMA ($\rho$ ranging from 0.74 to 0.85, *p*-value<0.001, details in Table 3), except for the Passive Range of Motion ($\rho$=0.58, *p*-value<0.001). Further, all task outcome measures were moderately to strongly correlated with the BBT (from $\rho$=0.54, *p*-value=0.0013 to $\rho$=0.77, *p*-value<0.001). None of the outcomes correlated significantly with the kUDT. For most tasks in the motor category there was a high level of agreement between subjects classified as impaired according to the FMA threshold and according to z-scores of the robotic metrics—Maximum Force Flexion, Active Range of Motion, as well as Maximum Velocity Flexion and Extension reached classification agreement of 80.00%, 83.33%, 76.67% and 80.00%, respectively. For Maximum Force Extension and Passive Range of Motion the agreement was lower—50.00 and 47.67%.

It was possible to distinguish between the affected side of stroke subjects and control subjects scores in the assessment of sensorimotor impairments (Tracking Error RMSE Slow: *p*-value<0.001, AUC=0.91 and Fast: *p*-value<0.001, AUC=0.92). Stroke subjects scored comparably to controls on their less affected side when following the slow trajectory (*p*-value=0.061; AUC=0.66), but significantly worse when following the fast trajectory (*p*-value=0.026, AUC=0.70). There was a significant difference between the affected and the less-affected side of stroke subjects (Slow: t-stat=5.40, *p*-value<0.001; AUC=0.81 and Fast: t-stat=4.82, *p*-value<0.001; AUC=0.80). 73.33% of stroke subjects were classified as impaired on their affected and 23.33% on the less affected side when following the slow trajectory. There was a moderate significant correlation between Tracking Error RMSE Slow and kUDT ($\rho$=$-0.44$, *p*-value=0.016), as well as a weak significant correlation between this metric and the BBT ($\rho$=$-0.39$, *p*-value=0.035), but it did not significantly correlate with the FMA. Tracking Error RMSE Fast was moderately correlated with the BBT ($\rho$=$-0.45$, *p*-value=0.014), as well as with the FMA ($\rho$=$-0.43$, *p*-value=0.017) and weakly with kUDT ($\rho$=$-0.39$, *p*-value=0.0347). Impairment classification according to the task z-scores was compared to both the kUDT and FMA, since this task was designed to involve both proprioceptive and motor function components. For both slow and fast trajectories, agreement between robotic z-score and the kUDT classification was 70.00%. When comparing the robotic z-scores classification to the one obtained with the FMA, Tracking Error RMSE Slow and Fast resulted in an agreement of 76.67 and 63.33%, respectively.

**Table 3:** Validity and feasibility results for all task metrics on the affected side

Category	Metric	AUC	% impaired	% class. agree.	Corr. FMA	Corr. BBT	Corr. kUDT	Time T1 [min]
Sensory	AE [*deg*]	0.95	66.67	70.00 (kUDT)	-0.32	-0.37*	-0.48*	3.52 ± 1.82
Motor	Vel. Flex. [*deg*/*s*]	0.91	60.00	76.67 (FMA)	0.74***	0.62***	-0.13	3.25 ± 0.55
	Vel. Ext. [*deg*/*s*]	0.94	90.00	80.00 (FMA)	0.76***	0.66***	-0.07	
	AROM [*deg*]	0.80	60.00	83.33 (FMA)	0.85***	0.77***	0.14	1.67 ± 0.46
	PROM [*deg*]	0.69	23.33	47.67 (FMA)	0.58***	0.56**	0.27	
	Force Flex. [*N*]	0.93	83.33	80.00 (FMA)	0.82***	0.69***	0.08	1.50 ± 0.89
	Force Ext. [*N*]	0.80	40.00	50.00 (FMA)	0.81***	0.73***	0.21	
Sensorimotor	RMSE Slow [*deg*]	0.91	73.33	70.00 (kUDT) $\mid$ 76.67 (FMA)	-0.33	-0.39*	-0.44*	3.80 ± 0.83
	RMSE Fast [*deg*]	0.92	46.67	70.00 (kUDT) $\mid$ 63.33 (FMA)	-0.43*	-0.45*	-0.39*	

*AE:* Position Matching Absolute Error, *Vel. Flex./Ext.:* Maximum Velocity Flexion/Extension,* AROM/PROM:* Active/Passive Range of Motion, *Force Flex./Ext.:* Maximum Force Flexion/Extension, *RMSE Slow/Fast:* Tracking Error RMSE Slow/Fast, *FMA:* Fugl-Meyer Upper Limb Motor Assessment, *kUDT:* kinesthetic Up-Down Test, *BBT:* Box & Block Test, *AUC:* Area Under the Curve of the Receiver Operating Characteristic, *% impaired:* % of stroke subjects classified as impaired according to z-scores, *% class. agree.:* % classification agreement as compared to clinical scores,* Corr.*: Spearman correlation of robotic metric with a clinical score. The defined threshold for discriminant validity was: AUC>0.7. Statistical significance: *p*-value<0.05: *, *p*-value<0.01: **, *p*-value<0.001: ***

### Independence of task metrics

Partial Spearman correlations between the five different robotic tasks are shown in Table 4. There was a moderate significant correlation between tasks within the motor category (Maximum Force Flexion, Active Range of Motion, Maximum Velocity Extension), $\rho$ reached 0.42, 0.45, 0.47 and *p*-values were equal to 0.030, 0.019 and 0.013. Further, there was a moderate significant correlation between Position Matching Absolute Error and Tracking Error RMSE Slow ($\rho$=0.50, *p*-value=0.007). No significant correlations were found between the proprioception assessment task and the tasks from the motor category. Likewise, tasks from the motor category were not significantly correlated with the assessment of sensorimotor impairments.

**Table 4:** Partial Spearman correlation coefficient $\rho$ between 5 different robotic task metrics on the affected side

		Motor			Sensorimotor
		Vel. Ext.	AROM	Force Flex.	RMSE Slow
Sensory	AE	0.32	− 0.25	− 0.11	0.50**
Motor	Vel. Ext.		0.47*	0.42*	− 0.20
	AROM			0.45*	− 0.02
	Force Flex.				0.09
Sensorimotor	RMSE Slow				

*AE*: Position Matching Absolute Error,*Vel. Ext.*: Maximum Velocity Extension,* AROM*: Active Range of Motion,* Force Flex.*: Maximum Force Flexion, *RMSE Slow*: Tracking Error RMSE Slow. Statistical significance: *p*-value<0.05: *, *p*-value<0.01: **

## Discussion

The aim of this study was to propose and validate a set of robot-assisted assessments of the hand, implemented on a 1-DOF robotic platform, in a group of 36 stroke and 31 control subjects. We demonstrated that the novel set of robotic task metrics has good to excellent clinimetric properties (reliability, discriminant and concurrent validity) and can independently inform of proprioceptive, motor and combined sensorimotor impairments. Taken together, the presented robot-assisted assessments provide quantitative insights into different aspects of sensorimotor function deficits at the level of the index finger MCP joint. The proposed robotic assessment approach might contribute to the understanding of patients’ impairment profiles and, in the future, allow to better track and predict recovery after neurological injuries, as well as personalize therapies.

### Test-retest reliability

Overall, the newly proposed robot-assisted assessments were reliable, did not show strong measurement error and were not confounded by strong systematic shift/learning effects. Demonstrating the reliability of the proprioception assessment (gauge position matching task) is an important contribution, since it is a long-standing challenge to reliably evaluate proprioceptive hand impairments in neurological patients, using either conventional or robotic methods [24, 36]. Good reliability was achieved while sampling each presented angle only once, which reduced overall assessment duration and hence contributed to clinical feasibility. The effect of angle magnitude on position matching error, which is in line with previous research [47, 72, 73], was consistent for all measurements, thereby not affecting reliability.

The reliability results obtained for the motor and sensorimotor task are in line with the ones previously reported in literature (ICC 0.7-0.9 for metrics of good quality) [12, 74–76], although it is challenging to directly compare these, as some studies were performed with control subjects instead of the target population. Compared to conventional clinical assessments, the proposed robotic assessments generally result in higher measurement error (e.g. FMA 10% [77], our robotic assessments between 15 and 25%). This could partially be explained by the precise sensing capability of the robotic method, which is more sensitive at detecting behavioural variability than clinical scales [76]. Given that the observed SRD% were relatively low, we could further hypothesize that, in the future, the metrics might be able to responsively capture longitudinal changes in impairments. The overall satisfying test-retest reliability results obtained in this study could be explained by the objective and sensitive nature of the measurement method. The robotic platform can deliver exact and repeatable stimuli and objectively measure the corresponding response, hence ensuring reproducibility of this method. Another factor contributing to the positive reliability results on the affected side is the large inter-subject variability of the stroke participants recruited in this study (severe to mild motor and proprioceptive impairments according to clinical assessments) [66]. Lower inter-subject variability and higher intra-subject variability could be observed on the less-affected side of stroke subjects, leading to overall lower intraclass correlation coefficients (although still within moderate to good reliability). This is because more subjects may score within the same range of values, however those values are more prone to some subject-specific confounds, such as mood, fatigue or motivation, since there is a wider spectrum of outcomes each subject can potentially achieve on their less affected side [66]. A systematic shift was detected for two robotic task metrics on the less affected side. Maximum Force Extension was on average lower at retest. This was likely influenced by three outliers (Additional file 1: Fig. SM6d), that might have had a slightly different finger positioning in the device at retest, which could have resulted in a lower fingertip force generated. The performance on the trajectory following task systematically improved on the retest on the less affected side. This task is the most challenging in the assessment battery in terms of motor skills, as it requires subjects to accurately follow a fast trajectory, which might explain the presence of some learning effect. Taken together, the positive reliability results achieved in this study lay out the foundation for integrating the robotic assessments in longitudinal studies to sensitively monitor the recovery of post-stroke subjects at the level of the hand.

### Discriminant validity

On the group level, stroke subjects performed significantly worse on the most affected side compared to age-matched control subjects in all robot-assisted assessments. This confirms that the robotic metrics capture abnormalities in task performance that can be related to specific post-stroke impairments. In addition, it is clinically relevant to provide information about existing impairments on a subject-by-subject basis. From the percentage of subjects classified as impaired, we see that not all stroke subjects scored worse than controls, depending on their impairment profile. In addition, from the graphical representation of different severity groups (Figs. 2d–4d & Additional file 1: SM1d-2d), a clear trend of decreasing task performance with increasing impairment severity can be observed. These results indicate the capability of the robotic assessments to discriminate between different impairment severities.

In more detail, according to Position Matching Absolute Error, 66.7% of stroke subjects were classified as impaired on their affected side according to the z-scores. This result is aligned with previous findings (robotic assessments 57-67% [22, 35], clinical—21–54% [21]). However, it should be noted that, in this work, we set an inclusion criterion to ensure that maximally 40% of recruited subjects had no proprioceptive deficits (as measured by the kUDT), hence the resulting prevalence might be higher than expected for an overall stroke subject population [15]. Position Matching Absolute Error was found to be significantly higher on the less affected side of stroke subjects than in control participants. Previous work has shown similar trends of proprioceptive deficits often occurring on both sides after stroke [35, 78]. Some of the possible physiological explanations for proprioceptive impairments being present on both sides include interhemispheric transfer of proprioceptive information [35, 79], as well as ipsilateral disinhibition due to central reorganizations after brain injury [80]. However, one should also consider that the gauge position matching task might, to some extent, be influenced by cognitive impairments, which could affect task performance on both sides. Indeed, we observed a weak significant correlation ($\rho$=0.386,*p*-value=0.0039, Additional file 1: Fig. SM8a) between the Position Matching Absolute Error (both sides) and MoCA (excluding patients presenting aphasia with MoCA<15, N=3), likely reflecting the cognitive requirements that are necessary to perform the task. In general, it is challenging to design a robotic assessment of proprioception that is completely free of confounds. Other existing approaches are influenced by attention deficits [36], memory [37], slow reaction time [35] or motor impairments [32]. However, in contrast to other work, we observed only a weak significant correlation in the Position Matching Absolute Error between the less and the more affected body sides ($\rho$=0.364, *p*-value=0.048, Additional file 1: Fig. SM8b). This suggests that our paradigm is only minimally confounded by cognitive impairments, as one would expect a stronger correlation between the body sides in case of a strong influence of cognitive impairments on the task outcome.

Secondly, it is interesting to observe that in the motor impairment assessments targeting finger extension (e.g. Maximum Velocity Extension), the difference between severely affected and moderately-mildly affected subjects was particularly noticeable and statistically significant in the case of AROM (Additional file 1: Fig. SM1d). Voluntary extension of the index finger is typically more impaired in the severely affected subjects [81], a measure which has been shown useful as a predictor of functional recovery and is hence recommended as a routine clinical assessment [45]. Therefore, the ability of our method to precisely capture limitations in finger extension early after stroke could aid clinical decision making. The percentage of subjects classified as impaired on their affected side varied between 23.3% and 90.0% depending on the motor task metric, which indicates that each task may be sensitive to a different aspect of a patient’s motor impairments. Similar trends were observed on another robotic platform, where depending on the task metric, different percentages of subjects were classified as impaired (19–81% [50]), despite the fact that over 80% of stroke subjects typically have some level of motor impairment [15, 16].

Finally, results of the sensorimotor impairment assessment are in line with previous findings indicating decreased upper limb/hand dexterity post-stroke [57, 82, 83]. However, such high-level group comparison analysis does not inform on the reasons for decreased performance in this task, i.e. whether subjects could not follow the trajectory due to weakness, impaired sensory feedback or both. A more refined picture can be obtained by considering severity subgroups, here created according to the clinical measure of proprioception (Fig. 4d). Some subjects within the group with no proprioceptive impairment as reported by the clinical test (kUDT = 2) showed high Tracking Error RMSE. This can be explained by the nature of this task, which not only measures proprioception, but the combination thereof with motor function. Therefore, these subjects most likely could not follow the trajectory due to weakness, although it is also possible that the process of integrating sensory input to generate motor output was affected [7]. This indicates that the results of the trajectory following task can be best interpreted when considered together with the two other categories of tasks, in order to understand the different components of the impaired performance.

### Concurrent validity

Moderate to strong significant correlations were found between the outcome measures of the tasks from each category (i.e. proprioceptive, motor, sensorimotor assessments) and their corresponding clinical scores, which indicates the capability of robotic metrics to capture specific impairments. In more detail, a moderate significant correlation was found between Position Matching Absolute Error and the kUDT, confirming that this robotic task is able to assess proprioceptive deficits. These observed correlations are in the expected range [12] given the limitations of the reference clinical score (ordinal scale, 0-2 points only [24, 36]). It would be of interest to further investigate concurrent validity using more accurate measures of somatosensory impairment, e.g. somatosensory evoked potentials [84], which precisely and objectively measure the strength and latency of somatosensory responses. Moreover, a weak significant correlation was found with the BBT, which may indicate the important role of proprioceptive feedback in the execution of dexterous activities of daily life involving the hand [14]. The fact that this robotic metric was not correlated with the FMA (motor subsection only, as the sensory subsection of FMA was not administered) suggests that this task might be able to assess proprioception independently of motor impairments. Further, Maximum Force, Active Range of Motion, as well as Maximum Velocity showed strong significant correlations with the FMA and no significant correlations with the kUDT. It is therefore likely that these robotic tasks reflect impairments in basic motor execution [13, 14]. In particular, we designed the fast target reaching task and its metrics to focus on feedforward control (rather than feedback, which could partially confound the motor outcomes in the presence of sensory impairments). Indeed, the peak velocity of the ballistic movement to the target occurred at 160 ± 57 ms from movement onset for stroke and 122 ± 22 ms for control subjects (average over all trials and subjects), which is a timeframe representative of feedforward control during movement execution [85, 86]. Given that the motor tasks correlated moderately to strongly with the BBT, it can be claimed that each of the kinematic and kinetic subcomponents of movement generation described by these robotic tasks (i.e. ability to displace the finger, generate force and fast motion), are necessary to produce the functional behaviour of grasping and holding an object. Finally, the trajectory following task resulted in slightly different correlations for the slow and fast movement trials. Slow trials follow a similar pattern to the position matching task—there is a moderate significant correlation between Tracking Error RMSE and the kUDT and weak significant correlation with the BBT. This would indicate that successful completion of this task primarily requires intact proprioceptive function. The lack of strong correlation with the FMA may indicate that this task relies on dexterity, which has been shown to be dissociated from basic motor function, like strength, for mildly to moderately impaired stroke subjects [14, 87]. Fast trials show moderate correlation to the BBT and the FMA, yet only a weak correlation with the kUDT, but all significant. It suggests that this task might integrate both motor and sensory components of movement execution. Stronger relation to motor scores in the fast trials can be explained by higher engagement of the motor system to generate faster movements, while slow movements require more dexterity. Overall, the proposed battery of assessments provides functionally relevant information about patients’ capabilities by dissecting different subcomponents of motor control (i.e. proprioception, strength, speed, sensorimotor function), which are necessary to execute activities of daily living (e.g. as in the BBT—grasping and lifting small objects).

### Independence of robotic task metrics

The fact that correlations between robotic assessments were at most moderate indicates that each task, to some extent, presents independent information. Interestingly, Tracking Error RMSE was correlated with Position Matching Absolute Error, but to none of the metrics in the motor impairment assessment category. Possibly this task requires fine motor control which is not needed in the simple motor execution-based tasks. Here the choice of the task metric for the sensorimotor task is crucial, given that if we chose, e.g., the range of motion during trajectory following instead of tracking error, the task outcome would have likely been correlated with motor metrics. However, we purposely chose Tracking Error RMSE by hypothesizing that this error-based metric could best describe the utilization of proprioceptive feedback in motor execution [57]. Both trajectory following and gauge position matching tasks assess some aspects of proprioception; one involves active (kinesthesia) and the other passive trajectory/position matching (position sense), hence some relationship between the two was expected [88, 89]. Although given the complex trajectory, the active following scenario resulted in higher errors. The lack of correlation between the tasks from motor and proprioceptive assessment categories indicated that these tasks, as desired, are capable of evaluating these types of impairments separately. This result is in line with previous findings, which highlighted the independence of motor and somatosensory deficits [33]. Overall, different tasks in the proposed battery of robotic assessments complement each other in providing a detailed characterization of each patient’s impairment profile at the level of the index finger MCP joint.

### Clinical feasibility

The protocol was completed by the majority of the recruited stroke subjects (only 2/36 subjects dropped out of the study and in total 30/36, that is 83% of recruited participants were included in data analysis) that had a broad range of different impairments (severe to mild impairments on the FMA and the kUDT). Hence, this assessment protocol is feasible to be performed by a majority of patients in the early stage of stroke rehabilitation, which could provide insights for designing appropriate therapy programs and predicting recovery. Further, the duration of the assessments was acceptable (recommended is less than 15 min per tested side [90]). However, taking together assessments of both sides, set-up, explanation and rest, the average session per patient was close to 1 hour, which is at the limit of feasibility for more severely affected patients. Reducing the number of trials in some of the tasks could help decrease the overall assessment time, only if such reduction doesn’t affect reliability of the task metrics. Analysis of the effect of trial number reduction on metric reliability will be done as future work in order to optimize the study protocol for prospective studies. Finally, we received positive feedback from the experimenters performing the assessments on the ETH MIKE, which is important, as their role is crucial in executing the robot-assisted assessments (instructing and assisting the patients throughout the protocol). The workload of the experimenters was reduced compared to performing standard clinical assessments, as many of the tasks were automatised (e.g. passive movement of the finger in the gauge position matching task). Therefore, the proposed assessments are clinically feasible also from the point of view of the clinicians operating the platform.

### Limitations

While the results of this study underline the ability of our metrics to identify specific aspects of hand sensorimotor impairments, several limitations need to be considered. The first limitation relates the device’s workspace constrained to $\pm 90^{\circ }$ (Fig. 1). From MCP joint neutral position, the setup allowed maximally $60^{\circ }$ flexion movement, which is smaller than full range of motion achievable by the index finger [91]. This particularly affected the fast target reaching task, in which some subjects overestimated their position with respect to the target and instead of naturally decelerating, were stopped by the device’s mechanical stop. That in turn affected reliability of that task, especially on the less affected side, where subjects were able to reach higher velocities. The solution is to expand the range of motion of the device by approx. $15^{\circ }$ for future studies. Another limitation concerns some of the task metrics. In the trajectory following task, subjects that could not move at all (N=4/30) scored better in Tracking Error RMSE than subjects that moved “randomly” (e.g. due to impaired proprioceptive feedback but good enough motor function to extend/flex the finger). This is not necessarily desired, because such a scoring system does not encourage subjects to do their best, since staying stationary may provide a better outcome. Similarly, in the gauge position matching task, subjects that always indicated the starting position as where they thought their finger was, because they did not perceive their finger position at all (N=2/30), scored better than those that tried to perform the task but were clearly off the target. These are shortcomings of how the metrics themselves are calculated. A possible solution could be to modify scores of subjects that did not move their finger or did not move the gauge indicator from the starting position, by e.g. assigning the worst possible score achieved by all other stroke subjects + 1 standard deviation, as has been done in other studies [33]. This method was not used in this study in order to allow analyzing clinimetric properties of the proposed metrics in their purest form, without the introduction of an arbitrary ceiling effect. As another limitation, it needs to be noted that hemispatial neglect or vision deficits were not systematically evaluated as a part of this study and could have had an effect on robotic task performance, since vision is crucial to correctly follow task instructions and perform the gauge position matching on the tablet. In future work, a detailed evaluation of hemispatial neglect e.g. with the Bell Test [92] should be added to the experimental protocol. Finally, this study only considered the index finger and it remains to be investigated how well these results generalize to the whole hand somatosensory and motor impairments. However, correlations of robotic motor assessments with clinical assessments targeting the whole upper limb observed in this study suggest that the index finger function is indeed essential for performing daily life activities (i.e., grasping objects as in the BBT) and it may be related to impairments in other parts of the upper limb (i.e., as shown through the strong correlations of some robotic tasks with the FMA), which is in agreement with findings from previous studies involving individual finger movements [93]. Previous research has reported high levels of agreement in somatosensory impairments of the same modality in adjacent body areas (especially hand and wrist) [17, 94]. This can potentially be explained by the presence of some interactions within representations of the neighbouring body areas in the primary somatosensory cortex [95]. It could therefore be expected that the results of our proprioception assessment may be translatable to other distal joints (e.g. other fingers). However, this would need to be verified in a dedicated study.

### Conclusions

In conclusion, this work successfully proposed and validated a set of robot-assisted assessments targeting proprioceptive, motor, and combined sensorimotor impairments in the hand. This contributes to addressing a long-standing gap in the neurorehabilitation domain, as such a comprehensive impairment profile of the hand could not be established in a reliable, valid, and clinically feasible manner before. Building a fine-grained picture of patients’ deficits is important to sensitively track rehabilitation progress and effectively adapt therapies. Generally, this work addresses a strong need for more sensitive, accurate and objective assessments, which could positively impact therapy planning and outcomes.

## Supplementary Information

**Additional file 1.**  Supplementary material. 

## Data Availability

The data presented in this manuscript are available upon reasonable request and under consideration of the ethical regulations.

## Abbreviations

D: Dominant
LA: Less affected
A: Affected
BBT: Box and Block Test
FMA: Fugl-Meyer Upper Limb Motor Assessment
kUDT: Kinesthetic Up-Down Test
ICC: Intraclass correlation coefficient
SRD: Smallest real difference

## Footnotes

1 https://www.drks.de/drks_web/navigate.do?navigationId=trial.HTML&TRIAL_ID=DRKS00018681

2 ETH MIKE video: https://youtu.be/jmWdwJ00onU
