# Supplementary material for: Reliable and valid robot-assisted assessments of hand proprioceptive, motor and sensorimotor impairments after stroke
Source: J Neuroeng Rehabil. 2021 Jul 16;18:115. doi: 10.1186/s12984-021-00904-5 (PMC8283922; doi:10.1186/s12984-021-00904-5)
Supplement: Supplementary file 1 — Additional file 1. Supplementary material. [file 12984_2021_904_MOESM1_ESM.pdf]

Reliable and valid robot-assisted assessments of hand proprioceptive, motor and sensorimotor impairments after stroke - **supplementary material**

Monika Zbytniewska, Christoph M. Kanzler, Lisa Jordan, Christian Salzmann, Joachim Liepert, Olivier Lambercy, Roger Gassert

30th of April 2021

Table SM1: Results of each task metric for control subjects (mean and standard deviation) and results of linear mixed effect modelling to find influence of confounds (p-values). Model moderate quality: C1 $\leq$ 15% and C2 $\leq$ 25%; good quality: C1 $\leq$ 10% and C2 $\leq$ 20%). Acronyms: SD: Standard Deviation; Dom: Dominant; Non-dom: Non-dominant.

**Category**	**Metric**	**Mean±SD**	**Mean±SD**	**Model quality**	**Age**	**Gender**	**Trial**	**Task-specific**	**Tested hand**
		Dom	Non-dom	C1(%), C2(%)	p-val.	p-val.	p-val.	p-val.	p-val.
Sensory	AE [deg]	5.17 ± 1.97	5.21 ± 2.71	11.72, 36.25	0.097	0.602	0.045	<0.001	0.608
Motor	Vel. Flex. [deg/s]	679.01 ± 166.96	589.48 ± 128.95	6.16, 19.40	0.945	<0.001	<0.001	<0.001	<0.001
	Vel. Ext. [deg/s]	435.93 ± 93.40	408.00 ± 76.20						
	AROM [deg]	76.77 ± 6.18	80.90 ± 8.85	7.30, 21.52	0.193	0.097	0.034	<0.001	<0.001
	PROM [deg]	88.63 ± 9.05	95.54 ± 11.18						
	Force Flex.[N]	38.55 ± 8.61	32.79 ± 10.93	6.76, 21.15	0.005	<0.001	<0.001	<0.001	<0.001
	Force Ext. [N]	9.26 ± 4.55	8.89 ± 4.51						
Sensorimotor	RMSE Slow [deg]	10.19 ± 4.13	9.35 ± 2.61	7.21, 23.31	0.104	0.497	0.103	<0.001	0.968
	RMSE Fast [deg]	10.85 ± 4.12	11.03 ± 2.92						

Table SM2: Test-retest reliability results for all task metrics: **less affected side**. Acronyms: AE: Position Matching Absolute Error; Vel. Flex./Ext.: Maximum Velocity Flexion/ Extension; AROM/PROM: Active/ Passive Range of Motion; Force Flex./Ext.: Maximum Force Flexion/ Extension; RMSE Slow/Fast: Tracking Error RMSE Slow/ Fast; T1: Test; T2: Retest; ICC: Intraclass Correlation Coefficient; SRD: Smallest Real Difference; SRD%: Smallest Real Difference as a percentage of the range of values across all trials; Shift: Systematic Shift; Correlation: Spearman Correlation between Test and Retest.p-value$<$0.05: *, p-value$<$0.01: **, p-value$<$0.001: ***.

Category	Metric	Mean±SD T1	Mean±SD T2	Mean±SD T2-T1	ICC(A,k)	SRD	SRD(%)	Shift	Correlation
Sensory	AE [deg]	12.08 ± 7.16	10.94 ± 6.28	3.93 ± 3.84	0.81 (0.77-0.83)	11.39	21.35	-4.01	0.60***
Motor	Vel. Flex. [deg/s]	399.89 ± 162.35	419.79 ± 159.91	19.89 ± 173.64	0.59 (0.38-0.73)	317.91	40.40	-3.20	0.44*
	Vel. Ext. [deg/s]	299.47 ± 87.09	315.00 ± 103.25	15.53 ± 75.97	0.81 (0.71-0.88)	116.75	22.22	-3.02	0.75***
	AROM [deg]	83.60 ± 12.79	81.27 ± 10.82	-2.34 ± 9.58	0.80 (0.70-0.87)	14.87	19.40	3.44	0.52**
	PROM [deg]	90.12 ± 8.14	90.53 ± 7.58	0.40 ± 6.61	0.79 (0.68-0.86)	10.38	19.36	-0.85	0.66***
	Force Flex.[N]	27.18 ± 13.35	27.50 ± 10.27	0.32 ± 8.49	0.86 (0.78-0.91)	12.65	24.92	-0.64	0.74***
	Force Ext. [N]	9.27 ± 5.16	7.90 ± 3.53	-1.38 ± 3.64	0.79 (0.68-0.86)	5.79	26.79	7.27	0.78***
Sensorimotor	RMSE Slow [deg]	12.58 ± 5.30	11.68 ± 4.25	-1.07 ± 3.91	0.83 (0.74-0.89)	5.76	23.38	-4.97	0.58***
	RMSE Fast [deg]	14.05 ± 5.05	12.68 ± 4.47	-1.37 ± 2.99	0.89 (0.82-0.93)	4.79	20.09	-7.24	0.53***

Table SM3: Validity and feasibility results for all task metrics: **less affected side**. Acronyms: AE: Position Matching Absolute Error; Vel. Flex./Ext.: Maximum Velocity Flexion/ Extension; AROM/PROM: Active/ Passive Range of Motion; Force Flex./Ext.: Maximum Force Flexion/ Extension; RMSE Slow/Fast: Tracking Error RMSE Slow/ Fast; AUC: Area Under the Curve of the Receiver Operating Characteristic; C: control; A: affected side; LA: less-affected side; paired t-test: reported values are [t-statistic, p-value]; % impaired: % of stroke subjects classified as impaired according to z-scores; T1: test.

Category	Metric	AUC (C vs LA)	AUC (A vs LA)	paired t-test (A vs LA)	% impaired	Time T1 [min]
Sensory	AE [deg]	0.82	0.61	[1.84, 0.077]	56.67	4.08 $\pm$ 1.75
Motor	Vel. Flex. [deg/s]	0.85	0.62	[1.93, 0.064]	60.00	3.33 $\pm$ 0.43
	Vel. Ext. [deg/s]	0.85	0.79	[5.17, 1.56e-05]	53.33	
	AROM [deg]	0.38	0.85	[6.51, 3.98e-07]	3.33	1.35 $\pm$ 0.29
	PROM [deg]	0.55	0.66	[3.67, 9.71e-04]	3.33	
	Force Flex.[N]	0.70	0.82	[5.79, 2.81e-06]	33.33	1.38 $\pm$ 0.29
	Force Ext. [N]	0.50	0.78	[5.19, 1.50e-05]	13.33	
Sensorimotor	RMSE Slow [deg]	0.66	0.81	[5.40, 8.37e-06]	23.33	3.95 $\pm$ 0.56
	RMSE Fast [deg]	0.70	0.80	[4.82, 4.12e-05]	20.00	

Table SM4: Demographic and clinical data per stroke subject recruited in the study. Acronyms: A: Affected side; LA: Less Affected side; FMA: Fugl-Meyer Upper Limb Motor Assessment; kUDT: kinesthetic Up-Down Test; BBT: Box& Block Test; MoCA: Montral Cognitive Assessment; MAS: Modified Ashworth Scale. There is missing data for subject 20, who dropped out of the study early, hence not all data was collected.

ID	Age	Gender	Affected side	Handedness	Weeks since stroke	Type of stroke	FMA	kUDT	BBT A	BBT LA	MoCA	MAS	Included?
1	71	Female	Left	Right	9.57	Ischemic	4	0	0	46	23	0	Yes
2	53	Male	Right	Left	6.43	Hemorrhagic	45	1	33	72	24	0	Yes
3	64	Male	Right	Right	4.71	Ischemic	19	0	4	50	7	0	Yes
4	73	Male	Left	Right	26.00	Hemorrhagic	36	0	11	50	19	0	No
5	70	Female	Right	Right	98.00	Hemorrhagic	32	0	23	54	23	0	Yes
6	43	Female	Left	Right	8.29	Hemorrhagic	64	2	42	73	23	0	Yes
7	64	Male	Right	Right	3.86	Ischemic	48	1	37	55	10	0	Yes
8	76	Male	Right	Right	4.86	Ischemic	5	2	0	55	19	0	Yes
9	81	Male	Right	Right	7.14	Ischemic	61	2	34	61	17	0	No
10	82	Male	Left	Right	3.86	Ischemic	4	0	0	51	25	0	No
11	66	Male	Right	Left	10.14	Ischemic	24	2	9	50	26	0	Yes
12	60	Female	Left	Right	13.29	Hemorrhagic	59	2	30	62	24	0	Yes
13	62	Male	Left	Right	5.00	Hemorrhagic	62	0	25	72	29	0	Yes
14	48	Male	Left	Right	5.71	Ischemic	65	1	74	80	28	0	Yes
15	63	Female	Left	Right	4.57	Hemorrhagic	4	0	0	69	25	0	Yes
16	29	Female	Left	Right	9.43	Hemorrhagic	19	0	2	63	25	0	Yes
17	70	Female	Left	Right	12.14	Ischemic	26	1	7	58	19	0	Yes
18	69	Female	Right	Right	8.14	Ischemic	60	2	60	72	22	0	Yes
19	49	Female	Left	Right	7.14	Ischemic	16	2	0	80	24	0	Yes
20	-	Female	-	-	-	-	2	1	-	-	18	0	No
21	75	Female	Left	Right	8.14	Ischemic	42	2	23	74	27	0	Yes
22	74	Male	Left	Right	14.71	Ischemic	37	2	30	44	27	0	Yes
23	76	Female	Right	Right	2.57	Hemorrhagic	63	2	41	37	18	0	No
24	57	Male	Left	Left	3.43	Ischemic	4	2	0	56	17	0	Yes
25	54	Male	Left	Right	20.86	Ischemic	47	0	17	57	30	0	Yes
26	81	Male	Left	Right	17.14	Ischemic	65	2	47	49	22	0	Yes
27	78	Male	Left	Right	4.43	Ischemic	16	2	0	51	20	0	Yes
28	68	Male	Right	Right	17.00	Hemorrhagic	40	2	29	61	28	0	Yes
29	80	Male	Right	Right	8.29	Ischemic	49	1	32	76	17	0	Yes
30	76	Male	Right	Right	2.86	Ischemic	4	1	0	47	6	3	Yes
31	63	Male	Left	Right	3.57	Ischemic	63	2	46	66	27	0	No
32	68	Male	Left	Right	6.71	Ischemic	4	1	0	45	20	0	Yes
33	29	Male	Right	Right	2.57	Hemorrhagic	63	2	26	48	28	0	Yes
34	71	Female	Right	Right	5.29	Ischemic	50	1	52	78	22	0	Yes
35	83	Male	Left	Left	7.14	Ischemic	56	0	25	56	22	0	Yes
36	84	Male	Left	Right	5.00	Ischemic	4	2	0	56	23	0	Yes

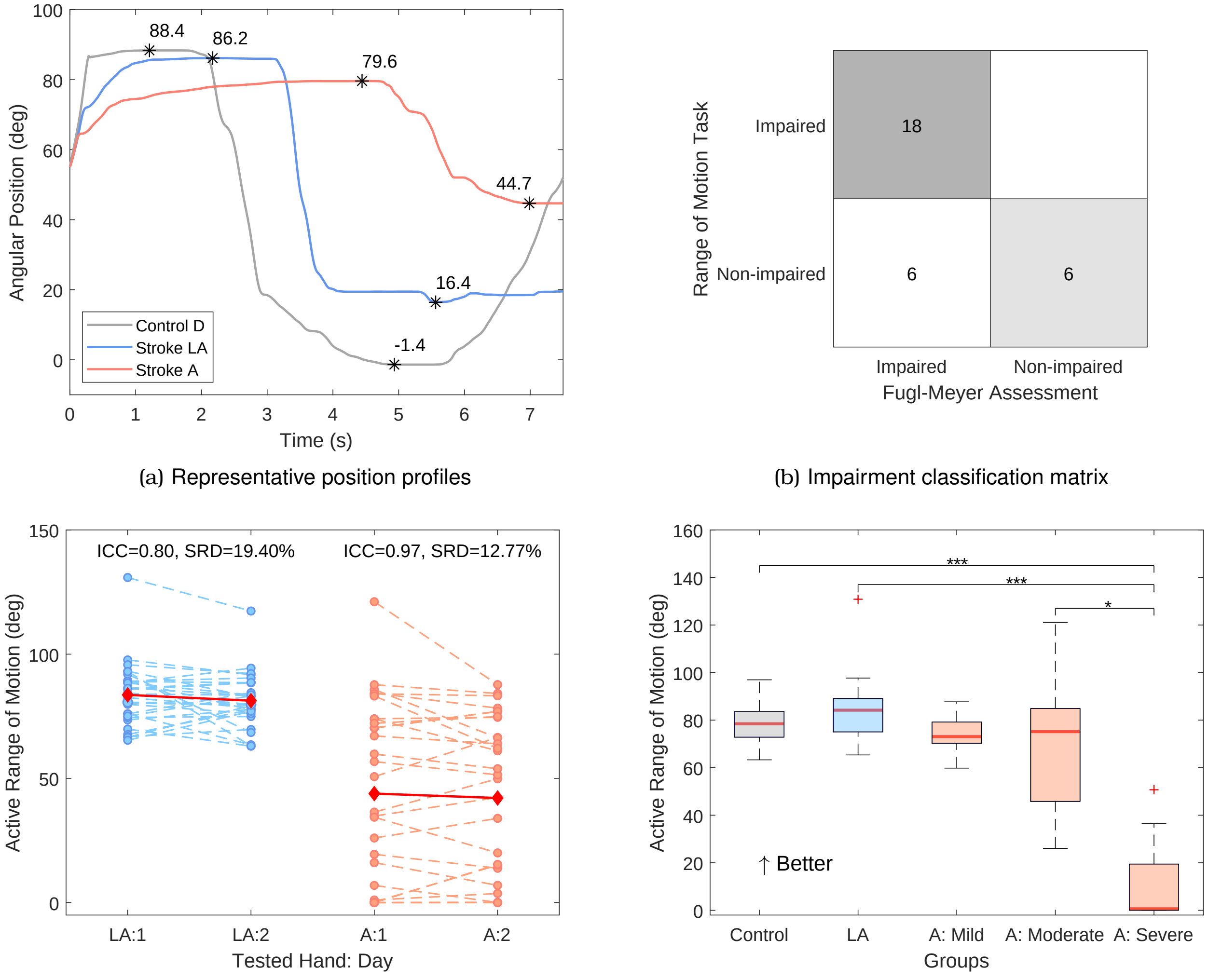

(a) Representative position profiles

(b) Impairment classification matrix

(c) Test-retest reliability

(d) Discriminant validity

Figure SM1: **Range of motion (active) task for the assessment of motor impairments.** a) Subjects are asked to move to maximum position in flexion and then in extension. The outcome measure is the difference in angular position between these two extremes. b) There is a high agreement (83%) in impairment classification between the task metric and the FMA. c) The task has good reliability (ICC$>$0.70) on the less affected side and excellent reliability (ICC$>$0.90) on the affected side. d) The box plot indicates a trend of decreasing performance with increasing stroke severity (mild: FMA$>= 54$, moderate: $54 >$FMA$>= 35$, severe: FMA< 35). There is a significant difference between controls (N=62) and severely impaired stroke subjects (N=14), as well as between moderately (N=8) and severely impaired stroke subgroups. Acronyms: D: dominant, LA: less affected, A: affected, FMA: Fugl-Meyer Upper Limb Motor Assessment, ICC: intraclass correlation coefficient, SRD: smallest real difference. Statistical significance: p-value$<$0.05: *, p-value$<$0.01: **, p-value$<$0.001: ***.

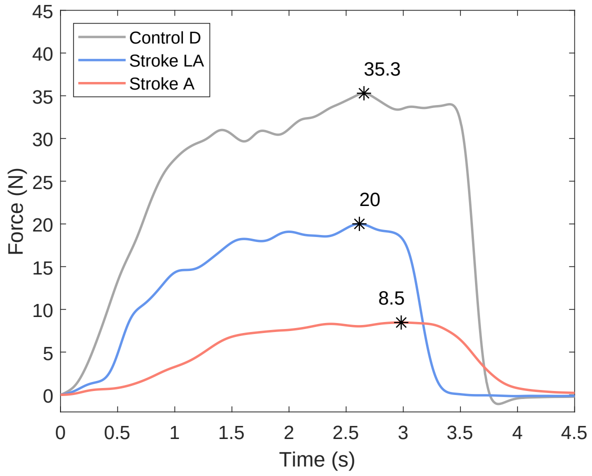

(a) Representative force profiles

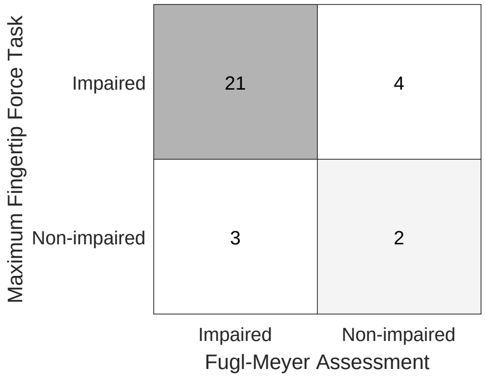

(b) Impairment classification matrix

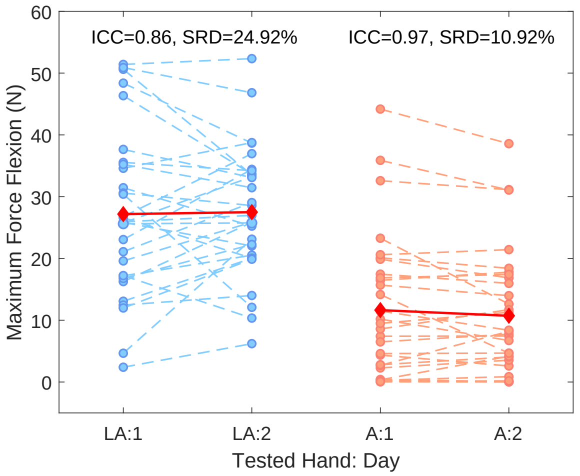

(c) Test-retest reliability

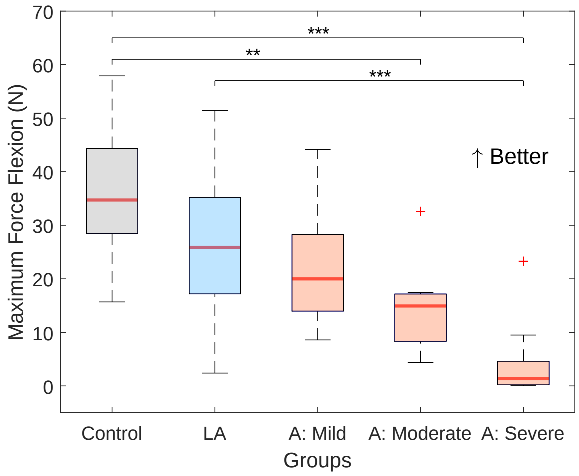

(d) Discriminant validity

Figure SM2: **Maximum fingertip force (flexion) task for the assessment of motor impairments.** a) Subjects are instructed to flex the finger as hard as possible against the fixated end-effector. Maximum fingertip force generated in flexion is the outcome measure for this task. b) There is a high agreement (80%) in impairment classification between the task metric and the FMA. c) The task metric has good reliability (ICC$>$0.70) on the less affected and excellent reliability (ICC$>$0.90) on the more affected side. d) The box plot indicates decreasing performance with increasing stroke severity (mild: FMA$>=54$, moderate: $54>$FMA$>=35$, severe: FMA<35). There is a significant difference between controls (N=62) and severely (N=14), as well as moderately (N=8) impaired stroke subgroups. Acronyms: D: dominant, LA: less affected, A: affected, FMA: Fugl-Meyer Upper Limb Motor Assessment, ICC: intraclass correlation coefficient, SRD: smallest real difference. Statistical significance: p-value$<$0.05: *, p-value$<$0.01: **, p-value$<$0.001: ***.

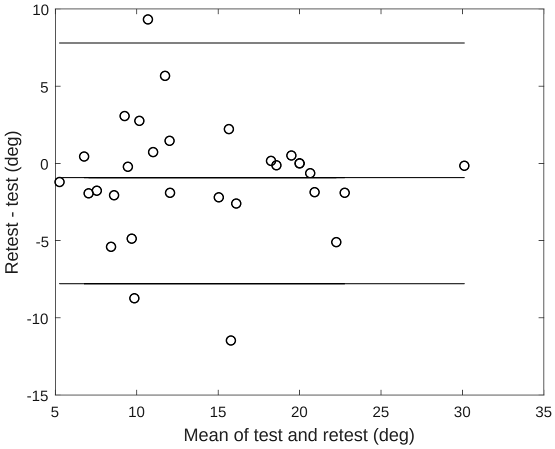

(a) Absolute Error; more-affected side

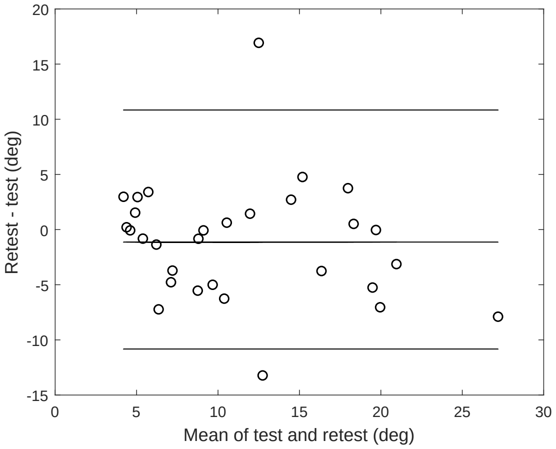

(b) Absolute Error; less-affected side

Figure SM3: **Gauge position matching task** (proprioceptive impairments assessment): Bland-Altman plots used to compare Position Matching Absolute Error on test and retest, as another measure of test-retest reliability. The plots indicate the presence of any systematic biases / learning effects between test and retest measurements. The middle horizontal line is the mean difference between test and retest, the two other horizontal lines are the limits of agreement.

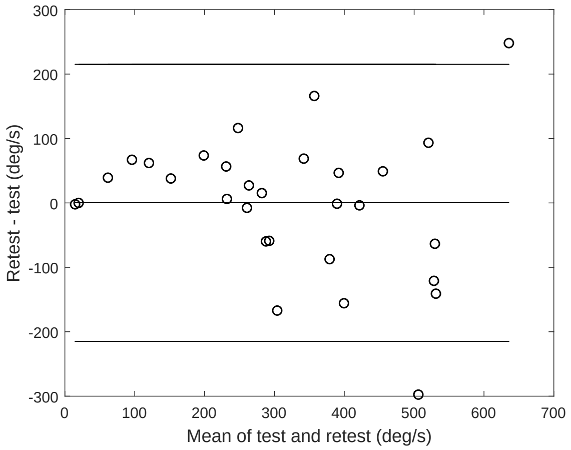

(a) Flexion; more-affected side

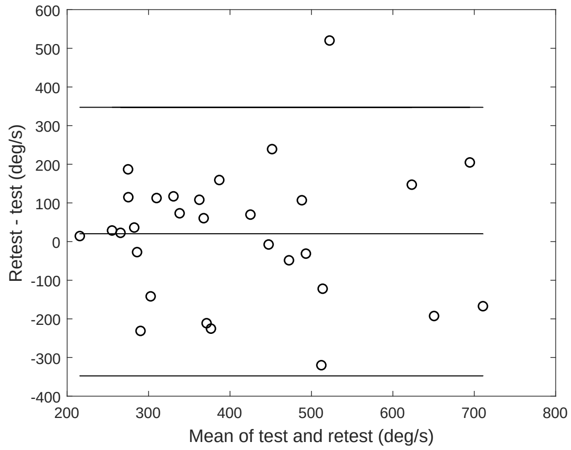

(b) Flexion; less-affected side

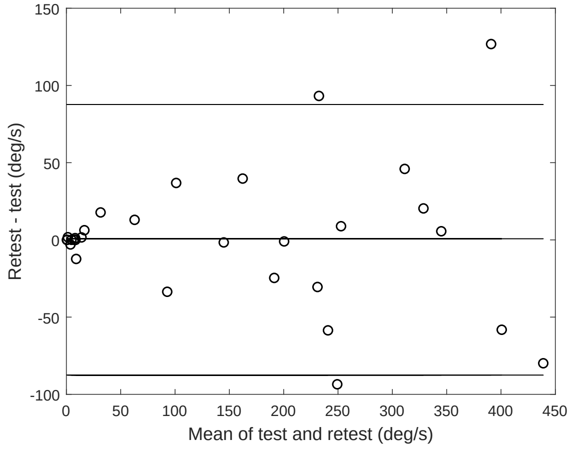

(c) Extension; more-affected side

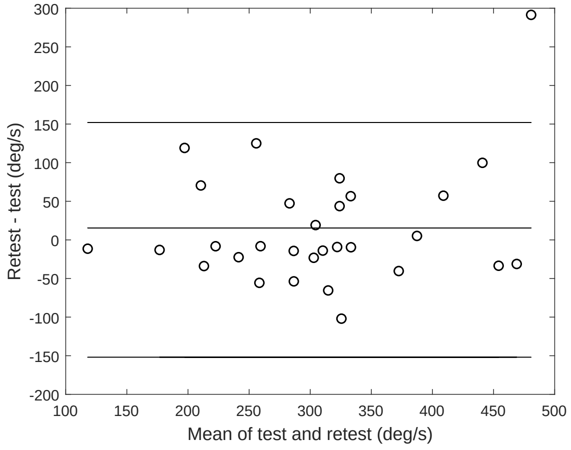

(d) Extension; less-affected side

Figure SM4: **Fast target reaching task** (motor impairments assessment): Bland-Altman plots used to compare Maximum Velocity (Flexion/Extension) on test and retest, as another measure of test-retest reliability. The plots indicate the presence of any systematic biases / learning effects between test and retest measurements. The middle horizontal line is the mean difference between test and retest, the two other horizontal lines are the limits of agreement.

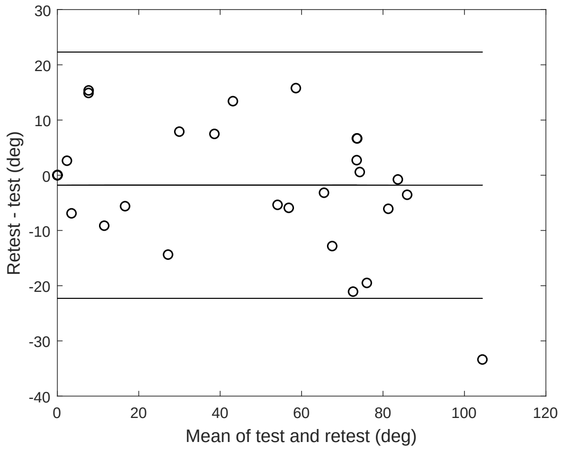

(a) Active Range of Motion; more-affected side

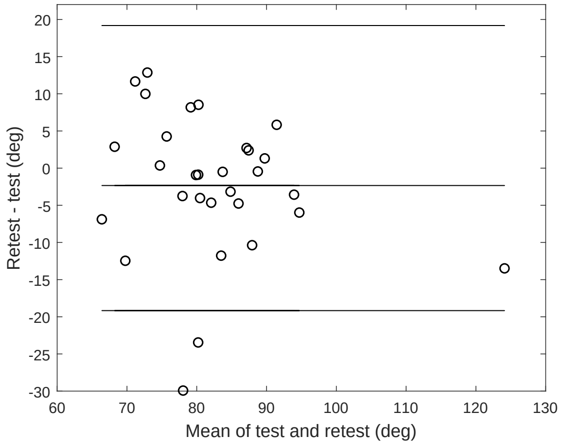

(b) Active Range of Motion; less-affected side

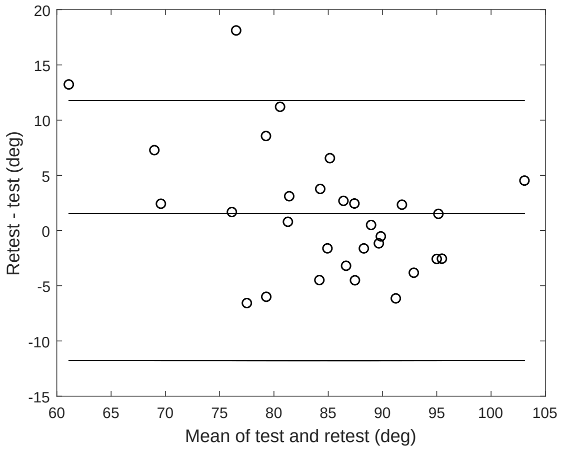

(c) Passive Range of Motion; more-affected side

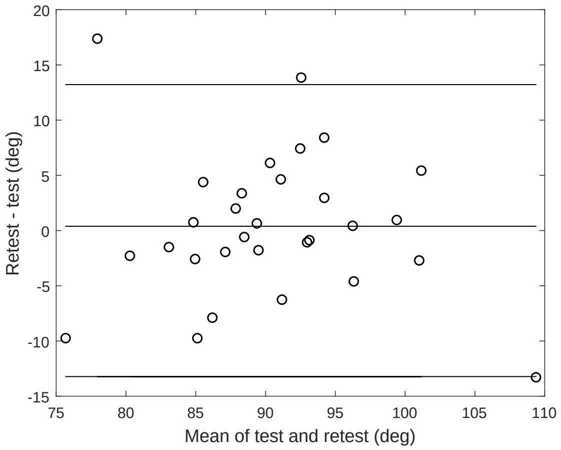

(d) Passive Range of Motion; less-affected side

Figure SM5: **Range of Motion task** (motor impairments assessment): Bland-Altman plots used to compare Active/Passive Range of Motion on test and retest, as another measure of test-retest reliability. The plots indicate the presence of any systematic biases / learning effects between test and retest measurements. The middle horizontal line is the mean difference between test and retest, the two other horizontal lines are the limits of agreement.

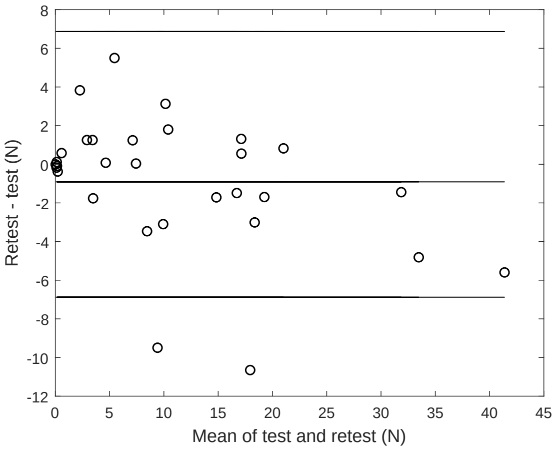

(a) Flexion; more-affected side

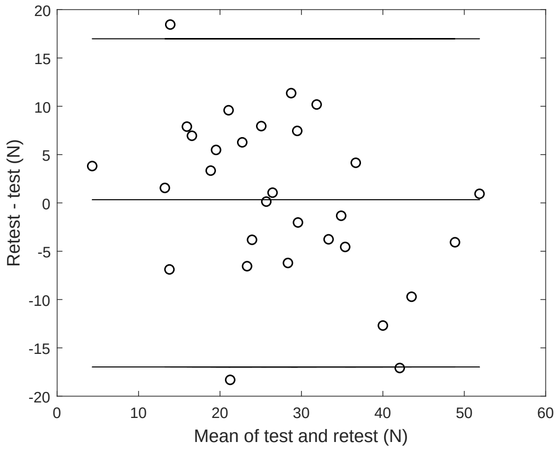

(b) Flexion; less-affected side

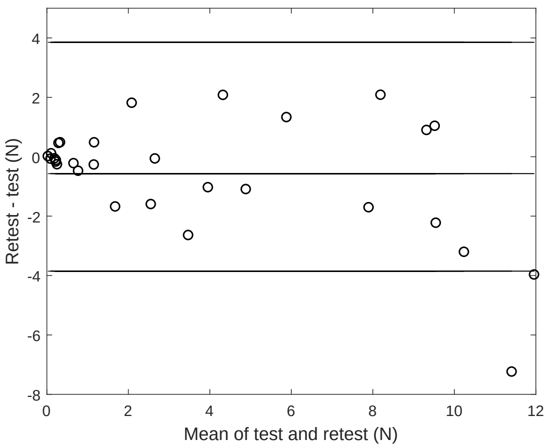

(c) Extension; more-affected side

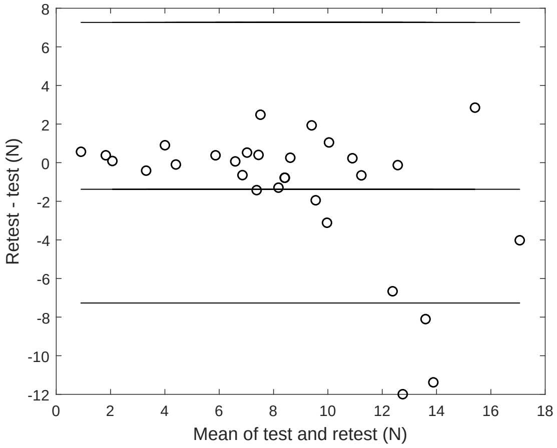

(d) Extension; less-affected side

Figure SM6: **Maximum fingertip force task** (motor impairments assessment): Bland-Altman plots used to compare Maximum Force (Flexion/Extension) on test and retest, as another measure of test-retest reliability. The plots indicate the presence of any systematic biases / learning effects between test and retest measurements. The middle horizontal line is the mean difference between test and retest, the two other horizontal lines are the limits of agreement.

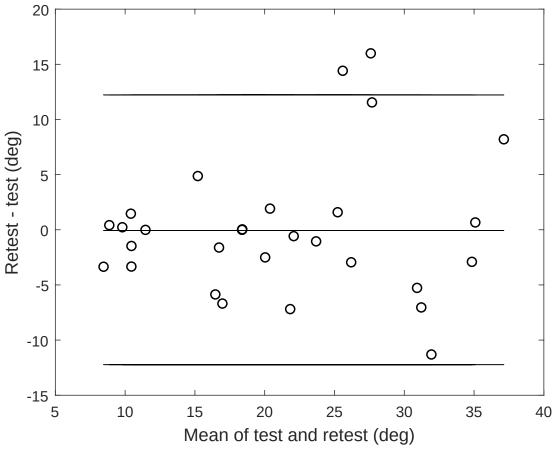

(a) Slow tracking; more-affected side

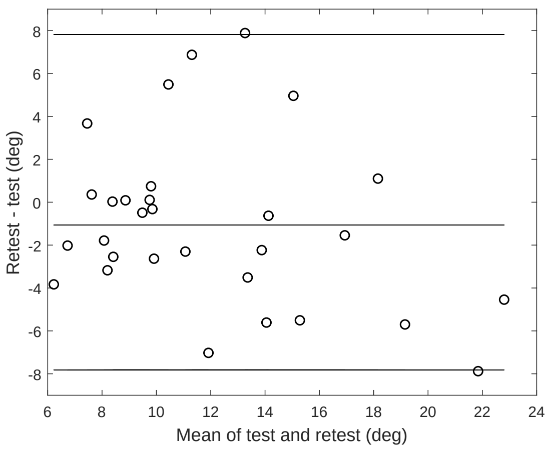

(b) Slow tracking; less-affected side

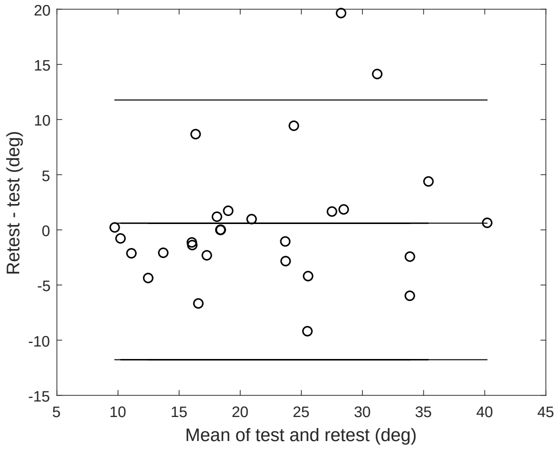

(c) Fast tracking; more-affected side

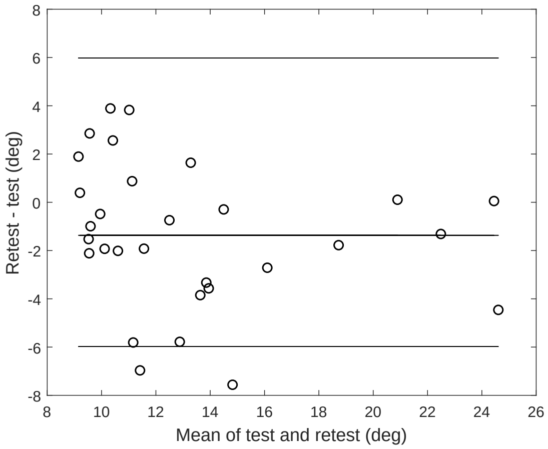

(d) Fast tracking; less-affected side

Figure SM7: **Trajectory following task** (sensorimotor impairments assessment): Bland-Altman plots used to compare Tracking Error RMSE (Slow/Fast) on test and retest, as another measure of test-retest reliability. The plots indicate the presence of any systematic biases / learning effects between test and retest measurements. The middle horizontal line is the mean difference between test and retest, the two other horizontal lines are the limits of agreement.

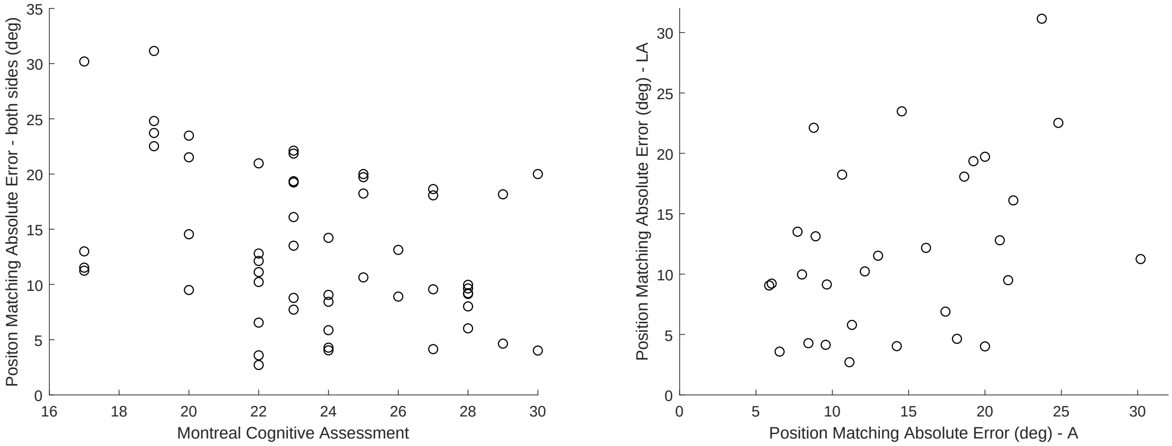

(a) MoCA vs Position Matching Absolute Error

(b) Less vs more affected side

Figure SM8: **Gauge position matching task** (proprioceptive impairments assessment): a) Correlation of Position Matching Absolute Error with Montreal Cognitive Assessment (Spearman ρ=0.386,p-value=0.0036). b) Correlation between the less and more affected side of stroke subjects (Spearman ρ=0.364,p-value=0.048). Abbreviations: A-affected side, LA-less affected side.
